# A novel RELA K119 deacetylation mediated by SIRT7 is a pivotal activator to exacerbate liver inflammation and fibrosis in teleosts

**DOI:** 10.1007/s42995-025-00287-9

**Published:** 2025-04-21

**Authors:** Xiaoliang Wu, Xiaofang Liang, Min Li, Jiacheng Liu, Chunyu Ge, Xiaoze Xie, Jie Wang, Yinhua Zheng, Hao Wang, Xiufeng Wu, Xu Gu, Min Xue

**Affiliations:** Institute of Feed Research, National Aquafeed Safety Assessment Center, Chinese Academy of Agricultural Sciences, Beijing, 100081 China

**Keywords:** Liver fibrosis, Inflammation, Acetylome, RELA, SIRT7, Deacetylation

## Abstract

Post-translational modifications (PTMs) regulate the activity and functionality of RELA, but their role in the pathogenesis of liver fibrosis is unclear. This study was performed to understand the regulation mechanism of acetylation of RELA on liver inflammation and fibrosis in a model animal of innate glucose intolerance, largemouth bass, and to provide a potential target and biomarker for liver fibrosis therapy. We found that the acetylation of total proteins and RELA was significantly reduced in fibrotic livers of largemouth bass induced by a high-carbohydrate and high-fat diet (HCHFD) and CCL4 challenge. Furthermore, quantitative acetylome data showed that the K119 site of RELA was deacetylated in fibrotic livers compared to healthy controls. Subsequently, we reveal a new mechanism that SIRT7 deacetylates RELA at the K119 site in largemouth bass. RELA K119 deacetylation enhances RELA transcriptional activity by increasing its DNA-binding activity, and facilitates nuclear translocation of RELA, resulting in the overwhelming release of proinflammatory factors, and subsequently enhancing liver inflammation and fibrosis. Pharmacological inhibition of SIRT7 using a specific inhibitor restores the decreased acetylation of RELA in vivo and in vitro, and reduces the transcriptional activity, nuclear localization of RELA and the expression of its target genes, which ultimately attenuates liver inflammation and fibrosis. These findings uncover a novel mechanism underlying liver fibrosis involving SIRT7-mediated deacetylation of RELA to activate the proinflammatory gene program, and thus provide important insights and biomarkers into the effective strategies for limiting liver inflammation and fibrosis.

**Supplementary Information:**

The online version contains supplementary material available at 10.1007/s42995-025-00287-9.

## Introduction

Nonalcoholic steatohepatitis (NASH) results in liver fibrosis, which is characterized by an excessive accumulation of extracellular matrix (ECM) in the hepatic tissue (Hernandez-Gea and Friedman 2011). Persistent liver fibrosis combined with chronic inflammation results in distorted hepatic architecture and loss of hepatocytes, ultimately impairing liver function (Alegre et al. 2017; Hung et al. 2022; Koyama and Brenner 2017). Durable inflammatory signals maintain the inflammatory hepatic environment by activating and recruiting immune cells to facilitate the progression of liver fibrosis (Tsuchida and Friedman 2017; Zhang et al. 2023). Thus, preventing chronic liver inflammation is a practical approach to stopping the development of hepatic fibrosis (Liu et al. 2020). Despite these advances, the precise mechanisms underlying liver inflammation and fibrosis, in particular when liver injury persists, remain elusive in humans and other species.

Largemouth bass is an economically important and widely distributed primary carnivorous freshwater fish species. To address the global crisis appertaining to the shortage of protein sources, a strategy for increasing the energy supply of affordable dietary carbohydrates in largemouth bass feed has been implemented (Gong et al. 2022; Wang et al. 2022). However, largemouth bass may spontaneously develop type 2 diabetes due to its severe intolerance of dietary carbohydrates, reflecting persistent hyperglycemia induced by a slow blood glucose clearance after ingestion (Biessels et al. 2010; Li et al. 2020). Liver fibrosis is a prevalent histologic phenotype, and is strongly associated with high-carbohydrate and/or high-fat diet consumption in largemouth bass and humans (Kohli et al. 2010; Wu et al. 2022; Zhong et al. 2022). The occurrence of metabolic disorders and liver fibrosis seriously limits the utility of dietary carbohydrates in the diet of largemouth bass. Moreover, recently largemouth bass has been regarded as an ideal animal model for studying glucose-induced metabolic liver disease (Chen et al. 2021a; Liu et al. 2022). However, the mechanism underlying carbohydrate-mediated liver fibrosis remains unclear in largemouth bass.

Post-translational modifications (PTMs), which underlie the functional diversity of proteins, are essential for homeostasis and pathologies (Karve and Cheema 2011). However, little is known about whether the cellular inflammation mechanisms mediated by PTMs are also involved in the pathogenesis of liver fibrosis. Nuclear factor kappa B (NFκB) is a key inflammation signal pathway in response to a variety of extracellular and intracellular stress (Liu et al. 2017). Phosphorylation of RELA promotes the recruitment of the co-activator CBP/p300, which acetylates RELA and ultimately enhances its transactivation of target genes (Buerki et al. 2008; Chen et al. 2005). In addition, acetylation of RELA at lysine 221 enhances RELA DNA-binding activity, leading to a prolonged RELA response in the nucleus (Chen et al. 2001). In contrast, acetylation of K122 and K123 decreases the DNA-binding activity and facilitates the export of RELA from the nucleus, resulting in a faster termination of the NF-κB response (Kiernan et al. 2003). Recent studies reported that RELA is also subjected to the regulation of O-GlcNAcylation, which impacts its transactivation via interplaying with phosphorylation and acetylation (Ma et al. 2017). Deacetylation of RELA mediated by deacetylase SIRT1, SIRT2, and HDAC3 also regulates NF-κB-dependent gene expression (Liu et al. 2014; Lu et al. 2022; Rothgiesser et al. 2010a). Collectively, the various modifications of RELA and the dynamic regulation of its associated enzymes endow RELA with versatile pathophysiologic functions under different conditions. However, the mechanism through which RELA protein modifications regulate liver fibrosis remains incompletely understood.

In this study, we uncover a novel mechanism in which SIRT7-mediated deacetylation of RELA at K119 promotes liver inflammation and fibrosis. Importantly, this effect may be rescued by a selective SIRT7 inhibitor. Our findings not only elucidate a previously unknown mechanism underlying liver inflammation and fibrosis but also provide important insights into the therapeutic approaches to target liver inflammation and fibrosis through restoration of adequate RELA acetylation.

## Materials and methods

This study was approved by the Ethics Committee of the Institute of Feed Research, Chinese Academy of Agriculture Sciences complying with all relevant ethical regulations (IFR-CAAS20220430).

### Animal models

Largemouth bass was purchased from a professional aquafarm (Tangshan, Hebei, China). All fish underwent two weeks of acclimation to laboratory conditions before the experiment, and were fed the control experiment diet. Three animal models were established in the present study. 1. Dietary inducing test. Fish (BW, 20–30 g) were fed a high-carbohydrate and high-fat diet (17.9% starch, 17.0% lipid, HCHFD) to induce hepatic inflammation and fibrosis, or a normal diet (9.4% starch, 12.1% lipid, ND) as a control for eight weeks. 2. Intense liver inflammation and liver fibrosis induction test. Fish (BW, 60–80 g) were randomly assigned to receive gavage of olive oil or carbon tetrachloride (CCl_4_ dissolved in olive oil at a ratio of 1:2, 100 μL/fish) three times per week for four weeks. 3. Acute liver inflammation inducing test. fish (BW, 20–30 g) were subjected to receive an intraperitoneal injection of 100 μL PBS or lipopolysaccharide (LPS diluted in sterile phosphate buffer (PBS), 5 mg/kg of fish). All fish were euthanized with an overdose of tri-chlorobutanol (300 mg/mL) before being used for sample collection.

## Experimental protocols

Experimental diets and aquarium facilities were prepared as described previously (Wu et al. 2022). Briefly, HCHFD and ND diets were processed into 3 mm diameter floating pellets under the following extrusion conditions: extrusion zone 1 (100 °C/5 s), extrusion zone 2 (110 °C/5 s) and die temperature (140 °C/4 s) using a twin-screwed extruder (YANGGONG MACHINE, China). Each diet was air-dried at room temperature, and stored at − 20 °C until use. The proximate composition analysis of the diets was exhibited in Supplementary Table S1. Fish were maintained in a recirculating aquaculture system (capacity: 256 L/tank) with a maximum of 30 fish per tank, and were fed to apparent satiation twice daily at 08:00 h and 16:00 h for four or eight weeks. During the growth period, the water temperature ranged from 23 ± 2 °C, dissolved oxygen (DO) > 6.2 mg/L, ammonia nitrogen levels < 0.3 mg/L and pH ~ 7.2. The photoperiod was 12 L:12 D, with the light period from 07:30 h to 19:30 h, and the light intensity was 400 lx.

### Tandem mass tagging (TMT) proteomics analysis

Normal and fibrotic liver tissues were harvested from the ND and HCHFD group, respectively. TMT-based proteomics and analysis was supported by Jingjie PTM BioLabs (Hangzhou, China), and experimental procedures of proteomics were performed as previously described (Wang et al. 2019). For protein extraction, the power of liver sample was added with lysis buffer (8 mol/L urea, 1% Protease Inhibitor Cocktail, acetylation inhibitor of 3 μmol/L TSA and 50 mmol/L NAM), followed by sonication three times on ice using a high intensity ultrasonic processor (Scientz). Finally, the supernatant was collected by centrifugation at 12,000 *g* at 4 °C for 10 min, and the protein concentration was determined with a BCA kit according to the manufacturer’s instructions. For trypsin digestion, 5 mmol/L dithiothreitol (56 °C, 30 min) and 11 mmol/L iodoacetamide (Room temperature, 15 min) was used to decrease and alkylate the sample solution, respectively. Then, 100 mmol/L TEAB was added to urea concentrations of less than 2 mol/L to dilute the protein sample. Next, the protein sample was digested firstly overnight at the mass rate of 1:50 trypsin-to-protein, and then secondly for 4 h at a 1:100 trypsin-to-protein mass ratio. Post-digested peptide was desalted and vacuum-dried, and then was reestablished using 0.5 mol/L TEAB, and processed according to the manufacturer’s instructions (TMT kit/iTRAQ kit). High pH reverse-phase HPLC (thermo Betasil C18 column, 5 μm particles, 10 mm ID, 250 mm length) was used to fractionate the tryptic peptides into fractions. Post-separated peptides were dissolved in NETN buffer, and subsequently incubated with pre-washed beads immobilized with Pan-acetylated antibody at 4 °C, overnight. After washing and eluting (0.1% trifluoroacetic acid), the eluted fractions were collected and vacuum-dried. For LC–MS/MS analysis, the dried peptides were desalted (C18 ZipTips, Millipore), dissolved in solvent A (0.1% formic acid) and directly loaded onto a homemade reversed-phase analytical column (15 cm length, 75 μm). Peptides were separated using a gradient on an EASY-nLC 1000 UPLC system. The peptides were subjected to an NSI source followed by tandem mass spectrometry (MS/MS) in Q ExactiveTM Plus (Thermo) coupled online to the UPLC. The Maxquant search engine was used to process the resulting MS/MS data. Tandem mass spectra were searched against the *Micropterus salmoides* NCBI database (Taxonomy ID: 27,706) concatenated with reverse decoy database. Trypsin/P was designated as the cleavage enzyme allowing up to 4 missing cleavages. The mass tolerance for precursor ions was set as 20 ppm in First search, and 5 ppm in Main search, and the mass tolerance for fragment ions was set as 0.02 Da. Carbamidomethyl on Cys was specified as fixed modification and acetylation modification, and oxidation on Met were specified as variable modifications. The FDR (false discovery rate) was adjusted to < 1% and minimum score for modified peptides was set > 40. Quantitative data with a *P* value < 0.05 was considered differential acetylation, Fold Change (FC) > 1.5 was taken as the threshold of significant up-regulation, and Fold Change (FC) < 1/1.5 was taken as the threshold of significant downregulation.

### SIRT7 inhibitor intervention

For in vitro treatment, primary hepatocytes were seeded in T25 culture flasks and grown to 80% confluence, followed by serum starvation. The cells were treated with a SIRT7 inhibitor (10 mol/L) for 12 h before being stimulated with LPS (200 g/mL) for an additional 12 h. For luciferase assays, 6 h after transfection, culture medium was discarded and fresh medium containing 10 µmol/L SIRT7 inhibitor was added for 24 h. For short-term SIRT7 intervention in vivo, SIRT7 inhibitor (4 mg/kg) was injected intraperitoneally into largemouth bass after 24 h, fasting for 24 h, and LPS was then injected intraperitoneally for an extra 24 h. For long-term SIRT7 intervention in vivo, the model of liver fibrosis was induced in fish by HCHFD. The baseline of liver fibrosis was decided and sacrificed at the initiation of SIRT7 inhibitor intervention in fish after eight weeks of HCHFD feeding without any treatment. Then, Fish was administered intragastrically SIRT7 inhibitor in a volume of 4 mg/kg or vehicle (the same volume of corn oil) four times a week for two weeks. Fish-fed ND without any treatment served as a blank control in this study.

### Primary hepatocytes isolation and culture

Primary hepatocytes were separated from largemouth bass (20–50 g), using the previously described protocol (Li et al. 2017) and modified as required. Briefly, MS 222 was used to anesthetize largemouth bass, which were then immersed in 70% ethanol for 2 min to sanitize the exterior surfaces. Liver tissues were excised aseptically and rinsed twice with precooled Hanks’ Balanced Salt Solution (HBSS) supplemented with streptomycin (100 μg/mL) and penicillin (100 IU/mL). The liver was then aseptically chopped into 1 mm^3^ pieces, after which it was digested in 0.125% sterile trypsin at 37 °C for 8 min. Using a 10 mL sterile pipette, the digested tissue was gently homogenized to form a cell suspension. Trypsin was neutralized with cell medium (Medium 199/ L-15 medium (V/V = 1:1), 10% FBS and 1% penicillin/streptomycin), followed by centrifugation at 1000 r/m for 5 min after which the supernatant was discarded. The cell sediment was resuspended in cell medium and filtered through a sterile 75 μm cell strainer, followed by low-speed centrifugation (1000 r/m, 5 min) and removal of the supernatant. Red blood cells were lysed for 3 min in precooled lysis buffer. The hepatocytes were cultured in cell medium containing Medium 199/L-15 medium (V/V = 1:1), 20% FBS, penicillin (100 IU/mL) and streptomycin (100 μg/mL), and then cultured in a humidified incubator at 27 °C.

### Plasmid construction and transfection

PCDNA3.1-RELA-HA overexpression plasmids were synthesized by Sangon Biotech (Shanghai, China). RELA mutants were generated using a site-directed mutagenesis kit (Vazyme Biotech). WT or mutants of RELA were inserted into PCMV-mCherry vector using a Cloning Kit (Vazyme Biotech). SIRT7 was amplified from a cDNA of liver tissue of largemouth bass and cloned into a PCMV-GFP vector. TNFα and IL-6 promoters were amplified from a DNA library of liver tissue and cloned into PGL6 plasmid (Beyotime) using a Super-Fidelity DNA Polymerase and Cloning Kit (Vazyme Biotech). All transfections were carried out using Lipofectamine 3000 (Life Technologies) in accordance with the manufacturer’s directions.

### Adenoviral infection of primary hepatocytes

Adenoviruses overexpressing RELA (WT, K119R) of largemouth bass were designed, validated and synthesized by Vigene Bioscience (Shandong, China). Primary liver cells were infected with adenovirus at a multiplicity of infection (MOI) 50. Ad-RELA WT or Ad-RELAK119R was incorporated into the culture medium when the cell confluency reached 70% for 48 h, followed either with or without LPS stimulation for the other 12 h. The negative control was an adenovirus recombinant encoding enhanced GFP (Ad-GFP).

### Nuclear extract preparation

The isolation of nuclear and cytosolic proteins from primary hepatocytes and fresh liver tissues were performed using commercial NE-PER™ Nuclear and Cytoplasmic Extraction Reagents (Thermo). The protein concentration was determined using a Quick Start^™^ Bradford Protein Assay Kit (Bio-Rad) according to the manufacturer’s instructions.

### Luciferase assays

The largemouth bass TNFα promoter (from − 2000 to + 100) and IL-6 (from − 2000 to + 100) were cloned into PGL6 vectors. Transient transfection and luciferase assays were separately performed using Lipofectamine 3000 (Life Technologies) and the Dual Luciferase Reporter Gene Assay Kit (Beyotime) according to the manufacturer’s instructions. Briefly, HEK293T cells were seeded in 24-well plates for 24 h before transfection. TNFα and IL-6 luciferase reporter plasmids, pRL (expressing renilla luciferase in all transfected cells) were co-transfected with RELA (WT, K119R, or K119Q) with or without the SIRT7 plasmid or empty into HEK293T cells. Cells were dissolved 24 h later, and the Dual-Luciferase Reporter Gene Assay Kit was used to detect luciferase activity. Renilla luciferase activity was used to normalize firefly luciferase activity.

### Electromobility shift assay (EMSA)

HEK 293 T cells were transfected with PCDNA3.1-RELA (WT, K119Q, or K119R)-HA constructs using Lipofectamine 3000 (Life Technologies). Nuclear proteins were obtained with the Nuclear and Cytoplasmic Protein Extraction Kit (Beyotime), and The Quick Start^™^ Bradford Protein Assay Kit (Bio-Rad) was used to measure the protein concentration. For each reaction, 8 µg of nuclear antigen was used. EMSA was carried out using the LightShift Chemiluminescent EMSA Kit (Thermo) in accordance with the manufacturer's instructions. The oligonucleotide probe for the TNFα promoters was labeled using an EMSA probe biotin labeling kit (Beyotime). Unlabeled competitors (wild-type and mutant) were added in 100-fold excess. Probe sequences are listed in Supplementary Table S2.

### RT-qPCR

TRIzol^®^ Reagent (Life Technologies) was used to extract total RNA from tissues and cells, and Fasting gDNA Dispelling RT SuperMix (TIANGEN) was used to generate first-strand cDNA according to the manufacturer's directions. The qPCR reaction was conducted with iTaq Universal SYBR Green Supermix (Bio-Rad) and the CFX96^™^ Real-Time System (Bio-Rad). Individual gene expression was normalized to the expression of EF1α. The RT-qPCR primers were listed in Supplementary Table S2.

### Co-immunoprecipitation

For cell immunoprecipitation, cells were seeded in six well plates and exposed to treatments for the experimental requirement. Lysis buffer for western blotting and IP (Beyotime) containing proteinase and phosphatase inhibitor cocktail (CST) was used to lyse the harvested cells; the lysis mixture was incubated on ice for 10 min. For liver tissue immunoprecipitation, fresh liver tissues were separated and aseptically minced into pieces, followed by homogenization to form cell suspensions in lysis buffer for western blotting and IP (Beyotime) containing proteinase and phosphatase inhibitor cocktail (CST), and incubated on ice for 20 min with mixing several times. Cell or tissue lysates were centrifuged at 4 °C for 15 min, and the supernate was incubated with the primary antibodies overnight at 4 °C. Then, the antigen–antibody complex was added into Protein A/G magnetic beads (Beyotime) washed three times with TBS for 2 h at room temperature with gentle rotation. The beads were rinsed three times with TBS. and eluted at 100 °C for 10 min with SDS sample buffer. For cytoplasmic and nuclear protein extract immunoprecipitation, cytoplasmic and nuclear extracts were separated stepwise from fresh liver tissues using NE-PER™ Nuclear and Cytoplasmic Extraction Reagents (Thermo) according to the manufacturer’s instructions. Prepared cytoplasmic and nuclear extracts were desalted by Zeba^™^ Spin Desalting Columns (Thermo), and then respectively incubated with the antibodies overnight at 4 °C. The antigen–antibody complex was added into Protein A/G magnetic beads (Beyotime) washed 3 times with TBS for 2 h at room temperature with gentle rotation. Beads were washed three times with TBS and eluted using SDS sample buffer at 100 °C for 10 min. Both the input and immunoprecipitates were subjected to western blot analysis, and the antibodies were listed in Supplementary Table S3.

### Immunofluorescence staining and the TUNEL Assay

For cell samples, HEK 293 T cells were seeded into Confocal Dishes and transfected with PCMV-RELA (WT, K119Q, K119R)-mCherry plasmids. Hoechst 33,342 (Beyotime) was used to stain the nuclei for living cells, and the fluorescence images were captured by Deltavision Ultra system (GE). For paraffin sections, these were deparaffinized and permeabilized (0.5% Triton X-100 in PBS) for 10 min, and subsequently blocked using QuickblockTM blocking buffer (Beyotime) for 10 min. They were then stained with rabbit polyclonal anti-α-SMA (1:100), and rabbit polyclonal anti-RELA (1:200) overnight at 4 °C. The sections were then rinsed three times and incubated for 1 h at room temperature with Alexa Fluor conjugated secondary antibodies (Life Technologies). Antifade mounting medium with DAPI (Beyotime) was added, and sealed with a coverslip. A TUNEL Apoptosis Assay Kit determined apoptosis signals in the liver according to the manufacturer’s instructions (Beyotime). After that, fluorescence images were collected using a Deltavision Ultra system (GE) and quantified by image J software (version: 1.4.3.67).

### Biochemical analysis

Hematological biochemical parameters, aminotransferase (ALT) and aspartate aminotransferase (AST), were analyzed using commercial reagents as per the manufacturer's instructions. (Nanjing Jincheng bioengineering). The levels of TNFα, IL-8, and IL-10 in liver tissues or cell culture supernatants were determined using commercial ELISA kits according to the manufacturer’s instructions (MEIMIAN).

### Histologic analysis

Liver tissues were fixed in 4% paraformaldehyde buffer for 24 h and embedded in paraffin. Liver fibrosis was assessed via Sirius red staining and Masson’s trichrome stain (Solarbio) of paraffin-embedded sections. The histologic features of the tissues were automatically observed and captured by TissueFAXS System (TissueGnostics) under a light microscope. The staining areas of Sirius red and Masson were quantified with StrataQuest software (TissueGnostics).

### Western blotting

Cells or tissues were collected and lysed in lysis buffer for western blotting and IP supplemented with a protease and phosphatase inhibitor cocktail (CST). Proteins were size fractionated by 12% SDS-PAGE and immunoblotted onto PVDF membranes (Millipore). The PVDF membranes with proteins were blocked with 5% BSA in TBST and sequentially incubated overnight at 4 °C with the indicated primary antibodies. HRP-conjugated AffiniPure Mouse Anti-Rabbit IgG Light Chain (abclonal) and Goat Anti-Rabbit IgG H&L (HRP) (abcam) secondary antibodies were used to incubate IP and input samples, respectively. The PVDF membranes were then detected using an ECL kit (Bio-Rad) and visualized in a ChemiDocTM XRS + with image LabTM System. Chemiluminescent signals were quantified by the Image J software (version 1.4.3). Signal intensities of targeted proteins were normalized with GAPDH and presented as fold changes. The antibodies used are listed in Supplementary Table S3. For RELA and TNFα specific antibodies of largemouth bass were synthesized and immunized in HUABIO.

### Statistics

SPSS18.0 statistical software was used to analyze the results, which were presented as the mean ± standard error of means (SEM). The charts were created by GraphPad Prism 7.0. No data were excluded during the data analysis. Shapiro–Wilk normality test was used to examine the normal distribution of samples. Student's t-tests (for data with a normal distribution) or Mann–Whitney *U*-tests (for data with a non-normal distribution) were used to determine statistical significance between two groups. One-way ANOVA corrected with Tukey’s multiple comparisons tests (with equal variance) or Dunnett’s T3 (with unequal variance) post hoc test was used to compare three or more experimental conditions, as indicated in each figure legend. All experiments were performed with at least three biological replicates as indicated in the figure legends. *P*-value < 0.05 was considered statistically significant (*P < 0.05, **P < 0.01, and ***P < 0.001).

## Results

### Lysine acetylation is decreased in liver inflammation and fibrosis in largemouth bass

**Fig. 1:**
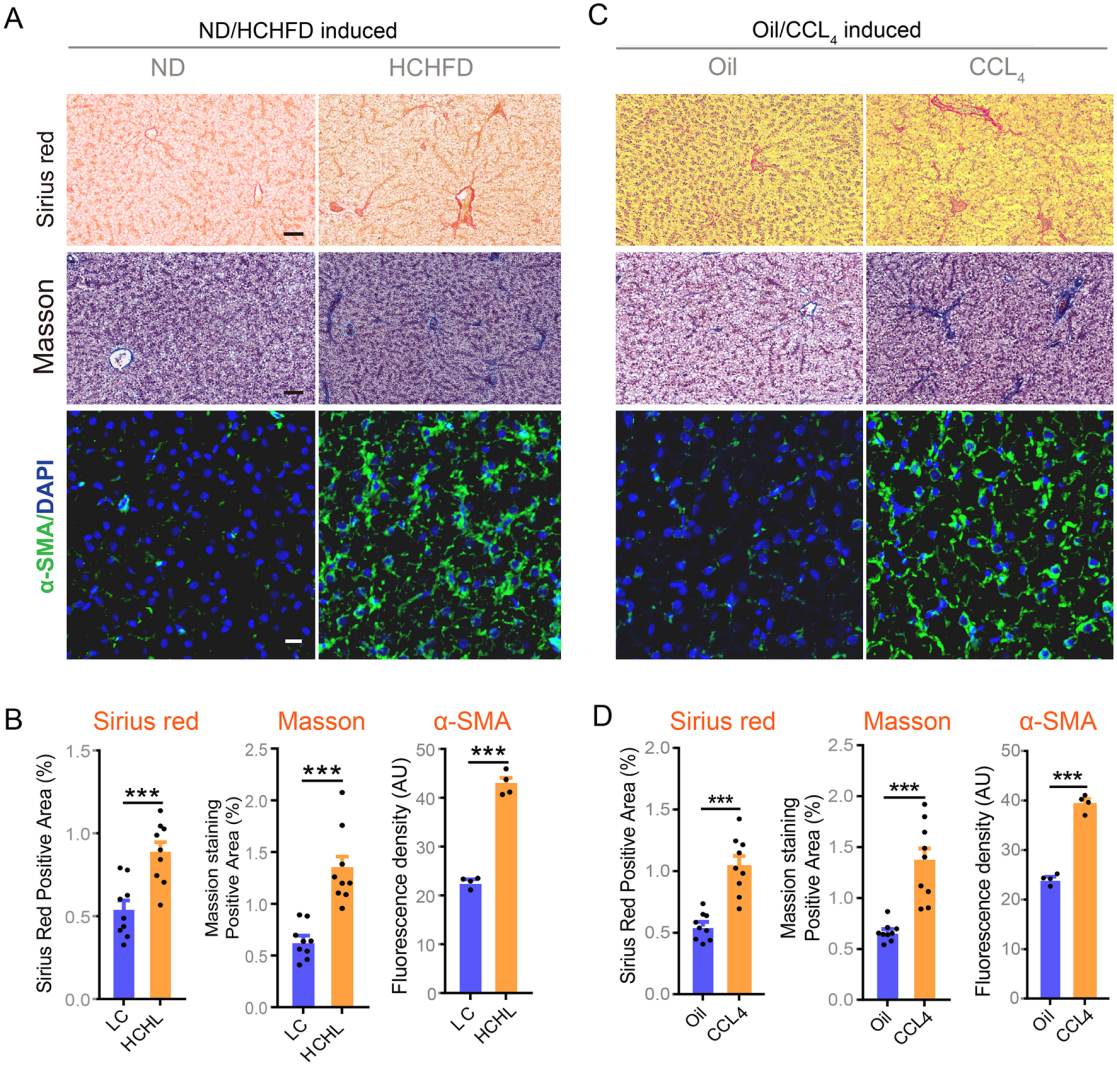
Lysine acetylation is decreased in HCHFD and CCL4-induced fibrotic liver and LPS-mediated inflamed liver. **A**, **C** Representative images of Sirius Red and Masson staining (*n* = 9), and representative immunofluorescence images of α-SMA for paraffin sections from livers induced by HCHFD (**A**) or CCL4 (**C**). Black scale bar represents 50 µm, White scale bar represents 20 µm for **A** and **C**. **B**, **D** The quantitation for Sirius Red and Masson staining and immunofluorescence staining of α-SMA as indicated. Data were presented as mean ± SEM. *P*-values were determined by two-tailed unpaired Student’s t-tests. *P < 0.05 was considered significant, **P < 0.01, ***P < 0.001

**Fig. 2:**
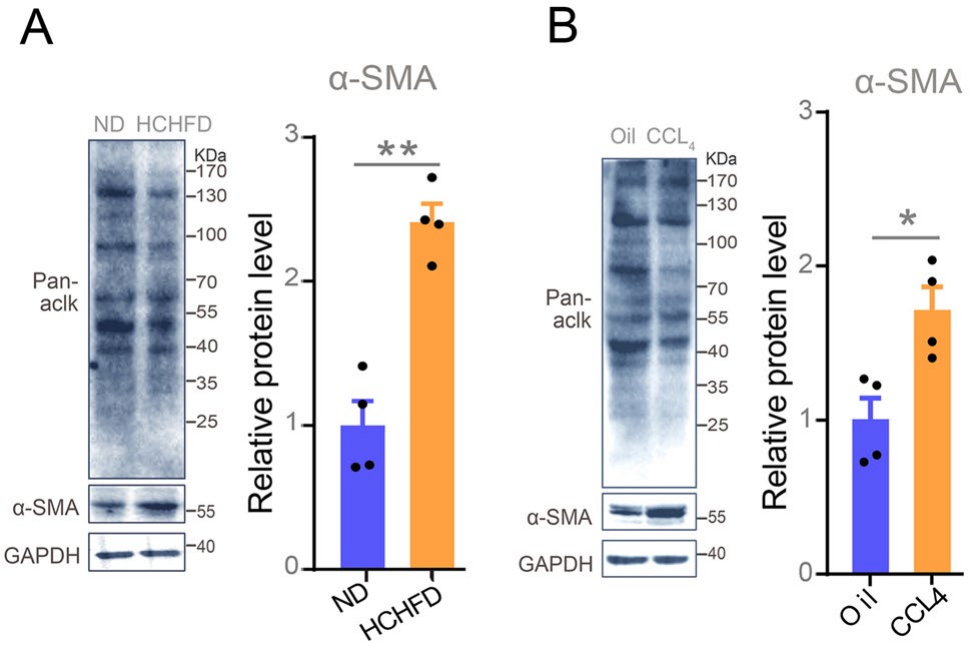
Representative western blot images of the total acetylation and α-SMA expression in the livers induced by ND versus HCHFD (**A**) or by oil versus CCL4 (**B**) and the respective quantitation of α-SMA (*n* = 4). Data were presented as mean ± SEM. *P*-values were determined by two-tailed unpaired Student’s *t*-tests. *P < 0.05 was considered significant, **P < 0.01, ***P < 0.001

To study the regulatory function of acetylation in liver inflammation and fibrosis in largemouth bass, we initially established fish models with acute liver inflammation induced by LPS and liver fibrosis mediated by a high-carbohydrate and high-fat diet (HCHFD) or CCL_4_ administration. Sirius Red, Masson’s staining, and immunofluorescence staining of α-SMA showed marked accumulation of collagenous fiber in fish livers fed with HCHFD diet (Fig. 1A, B) or challenged with CCL_4_ (Fig. 1C, D). Significantly elevated protein levels of α-SMA (Fig. 2A, B), a gold standard fibrosis marker, and mRNA levels of fibrotic genes, such as *Acta2, Col1α1, Col1α2, Col2α, Mmp2, and Mmp9*, as well as proinflammatory genes, i.e. *TNFα, IL-8, IL-10* and *IL-1β,* further illustrated the occurrence of liver fibrosis in fish fed HCHFD diet (Supplementary Fig. S1A, B) or challenged with CCL_4_ (Supplementary Fig. S1C, D). These data support that the pathological phenotype of liver fibrosis in largemouth bass was successfully established. Interestingly, we observed a significant reduction in amount of acetylated protein in liver from HCHFD-fed and CCL_4_-challenged fish revealed by western blot (WB) analysis (Fig. 2A, B). Next, we determined whether there was an abundance of acetylated protein in liver with acute inflammation. Thus, significantly elevated mRNA levels of proinflammatory genes, such as *TNFα, IL-8, IL-10* and *IL-1β,* and protein levels of TNFα, IL- and IL-1β indicated that liver inflammation in largemouth bass was induced by LPS (Supplementary Fig. S2A–C). In line with the results from liver fibrosis, the amount of acetylated protein was also reduced in inflamed livers induced by LPS, as revealed by western blot analysis (Supplementary Fig. S2D).

These data demonstrated that decreased acetylation is associated with liver inflammation and fibrosis in largemouth bass, suggesting that acetylation may govern liver inflammation and fibrosis in response to HCHFD diet or damage by chemical reagent.

### RELA K119 acetylation is decreased under conditions of liver inflammation and fibrosis

**Fig. 3:**
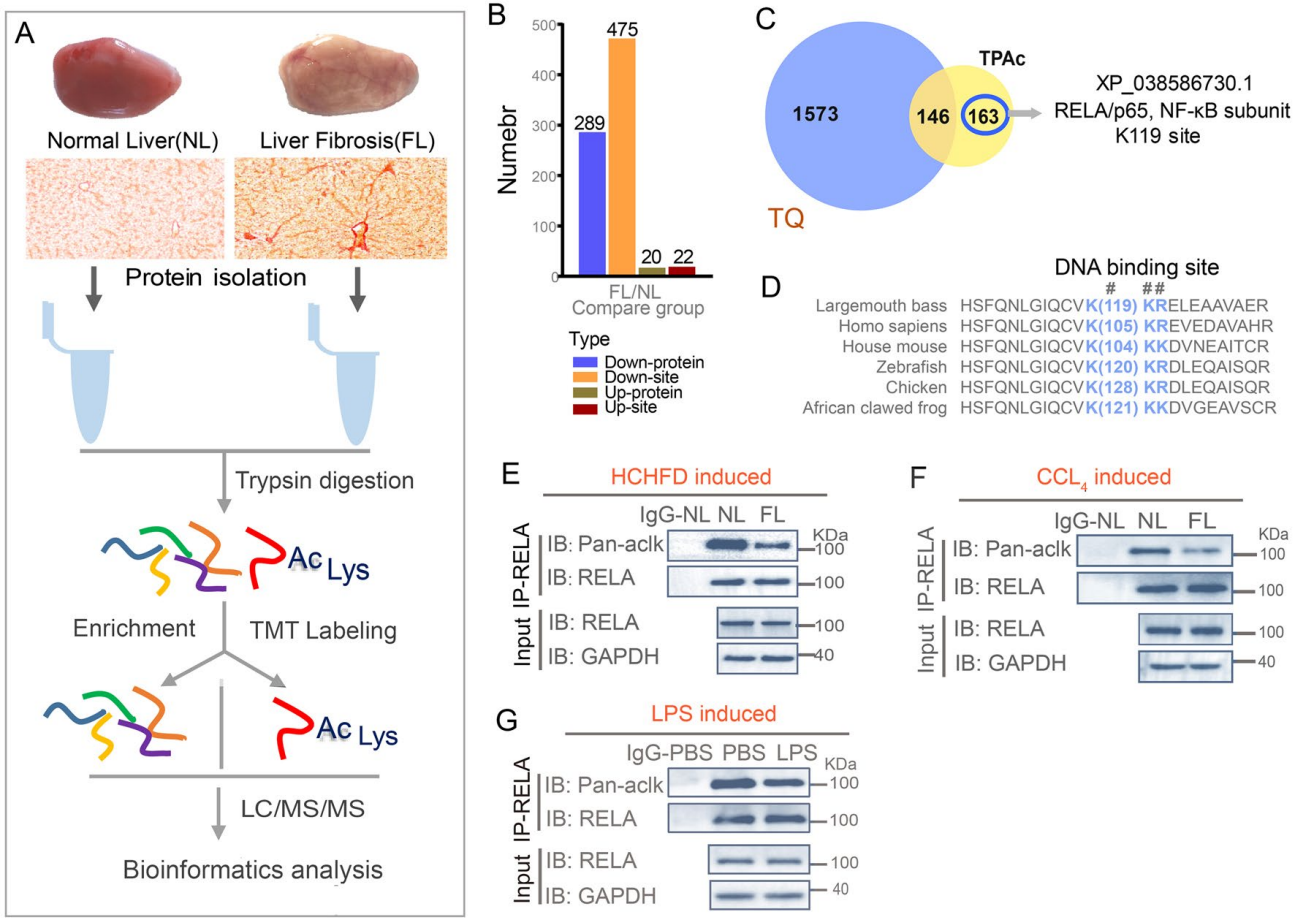
Identification of RELA-K119 acetylation in fibrotic livers through acetylome screen. **A** Schematic of strategy for identification and quantification of acetylome between normal liver (NL) and fibrotic liver (FL) (*n* = 3). **B** The number of differentially modified sites and acetylated proteins between normal and fibrotic liver. **C** 163 acetylated proteins without changes in total protein levels were revealed by Veen analysis between acetylome and total proteome. **D** RELA K119 resides in a conserved DNA-binding site in Rel homology domain (RHD). Lysine 119 of RELA was highlighted in luminous yellow. **E**–**G** Total protein extracted from normal or fibrotic livers induced by ND versus HCHFD (**E**) and by oil versus CCL4 (**F**) and livers treated with PBS versus LPS (**G**) was subjected to immunoprecipitation of RELA followed by western blot of Pan-aclk. Representative western blot images were shown (*n* = 3)

To further determine the role of acetylation in hepatopathy and identify key regulators of liver inflammation and fibrosis, we combined Tandem Mass Tags (TMT) labeling using a highly specific anti-acetyl lysine antibody and LC–MS/MS analysis to systematically assess the acetylome in normal and HCHFD-induced fibrotic livers (Fig. 3A). A total of 497 differentially acetylated sites in 309 proteins were identified, including 475 sites in 289 proteins with decreased acetylation, and 22 sites in 20 proteins with increased acetylation (Fig. 3B). Hierarchical clustering analysis revealed that the differentially modified sites could be effectively distinguished between normal (NL1-3) and fibrotic liver samples (FL1-3) (Supplementary Fig. S3A). To focus on the proteins only with changes in acetylation but not in total protein level, we performed the Venn analysis of previously published differential expression proteins in total proteomics data (Wu et al. 2022) and 309 proteins from our acetylome screen. Thus, 163 acetylated proteins without changes in total protein levels were revealed (Fig. 3C). Interestingly, these proteins were involved mainly in molecule binding based on molecular function analysis (Supplementary Fig. S3B). Among these proteins, RELA, a subunit of nuclear factor kappa B (NF-κB) caught our attention given its diverse functions in inflammation, disease, stress response (Supplementary Dataset 1) and established roles in NASH in mammals (Chen et al. 2021b). As shown in Supplementary Fig. S4, RELA was deacetylated at a single lysine, Lys119 (K119ac). This site is within the Rel homology domain (RHD), a conserved DNA-binding site from largemouth bass to *Homo sapien*s (Fig. 3D).

To further determine whether RELA was deacetylated in fibrotic and inflamed livers compared to normal livers, we performed immunoprecipitation of endogenous RELA in total proteins extracted from largemouth bass livers and followed by western blot analysis using a pan-aclk antibody. As shown in Fig. 3 E, F, the acetylation of RELA was significantly reduced in fibrotic liver induced by either HCHFD or CCL_4_, and in inflamed liver mediated by LPS (Fig. 3G) compared with the respective controls. Then, we evaluated whether RELA acetylation was also downregulated in primary hepatocytes stimulated by LPS (Supplementary Fig. S5A). Thus, after LPS stimulation, the mRNA levels of RELA target genes, such as *TNFα, IL-8, IL-10, IL-1β, Mmp9, Bcl-2* and *Bax*, and secretion of proinflammatory factors TNFα, IL-8, IL-1β were significantly enhanced (Supplementary Fig. S5B, C). In contrast, the acetylation of RELA was dramatically reduced (Supplementary Fig. S4D).

Taken together, these results show that RELA is deacetylated in fibrotic and acute liver inflammation in vivo, and in LPS-stimulated primary hepatocytes in vitro. This supports the notion that increased expression of RELA target genes is associated with the decreased acetylation of RELA.

### Deacetylation of RELA K119 enhanced its transcriptional activity and target gene expression

**Fig. 4:**
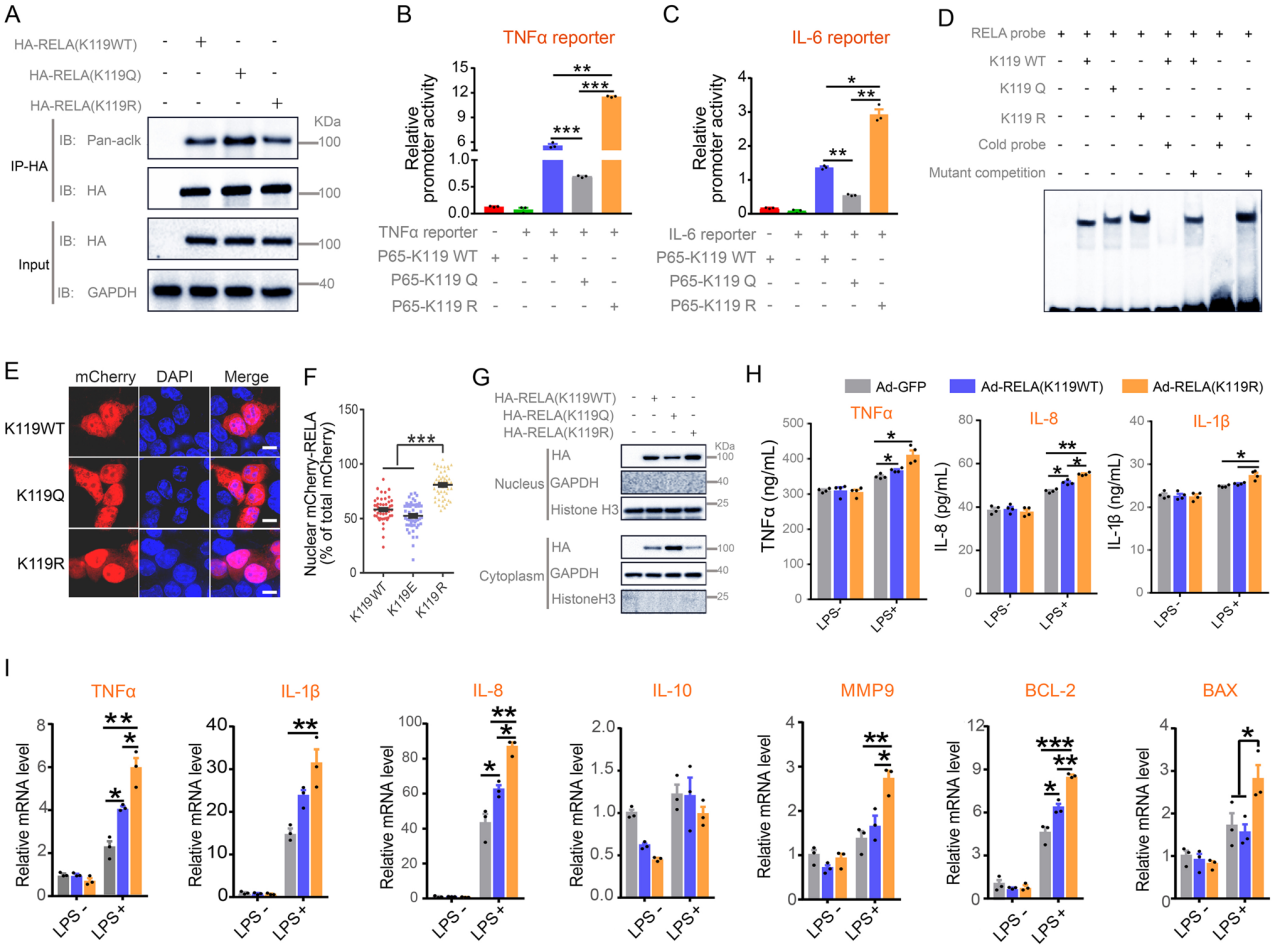
RELA deacetylation enhances its transcriptional activity and expression of target genes. **A** Representative western blot images for immunoprecipitation of HA followed by western blot of the indicated proteins in HEK 293 T cells transfected with RELA K119WT, K119Q or K119R expression plasmid for 24 h. **B**, **C** Luciferase assay for effects of WT RELA and the indicated RELA mutants on the promoter activity of TNFα (B) and IL-6 (**C**) in HEK293 cells. **D** Representative image of Electromobility shift assay (EMSA) under the indicated conditions. Specific binding was validated by the indicated WT and mutant probes. **E** Representative fluorescence image for subcellular localization of RELA in HEK293T cells transfected with mCherry-tagged K119WT, K119Q, K119R expression plasmids. Scale bars for E, 8 µm. **F** Quantitation of the nuclear versus total RELA for panel E (*n* = 50 cells per group). **G** Representative western blot images for subcellular distribution of exogenous HA-tagged RELA K119WT, K119Q or K119R proteins in HEK293T cells. GAPDH and Histone H3 were included as positive control for cytosolic and nuclear protein, respectively. **H** ELISA analysis of the protein levels of the indicated proinflammatory factors in the supernatant of primary hepatocytes transduced with Adenovirus carrying RELA K119WT (Ad-RELA K119WT) or RELA K119R (Ad-RELA K119R) transcript versus Ad-empty control (Ad-GFP) for 48 h followed by LPS stimulation for 12 h. I qRT-PCR analysis of the indicated RELA target genes in primary hepatocytes transduced with the indicated Adenovirus for 48 h followed by the stimulation of LPS or vehicle control for 12 h. Data were presented as mean ± SEM. *n* = 3 independent experiments for **A**, **B**, **C**, **D**, **E**, **F**, **G**, and **I**; *n* = 4 independent experiments for H. *P*-values were determined by one-way ANOVA corrected with Tukey’s multiple comparisons test in Fig. 4F, H, I and corrected with Dunnett’s T3 post hoc tests in Fig. 4B, C. *P < 0.05 was considered significant, **P < 0.01, ***P < 0.001

To further determine the acetylation of RELA K119, we mutated K119 (WT) to arginine (R) (mimicked the deacetylated state of protein) and glutamine (Q) (mimicked the hyperacetylated state of protein), respectively. As shown in Fig. 4A, the K119Q mutant demonstrated increased acetylation compared to RELA wild type (WT), whereas K119R displayed an opposite trend. These data further validate the importance of K119 in the acetylation of RELA in largemouth bass. Although RELA is a well-known transcription factor and its acetylation in specific lysine residues affects both DNA-binding ability and transcriptional activity of the protein in mammals have been reported (Yeung et al. 2004), the relevant function of RELA in largemouth bass is unclear. TNFα and IL-6 are well-characterized RELA target genes in humans and mice. Next, we obtained the promoter sequences of TNFα and IL-6 of largemouth bass from the National Center for Biotechnology Information (NCBI) website (https://www.ncbi.nlm.nih.gov/), and the binding sites for RELA were predicted by the JASPAR database (https://jaspar.genereg.net/) to perform the luciferase assay. As shown in Supplementary Fig. S6A and C, multiple RELA binding sites were predicted in both TNFα (A) and IL-6 (C) promoter regions. To determine the functionality of these predicted sites in RELA-activated TNFα and IL-6 gene transcription in largemouth bass, we mutated these predicted sites individually and performed a luciferase assay. Robust induction of luciferase activity of WT TNFα and IL-6 reporter mediated by RELA was observed, whereas such induction was diminished in mutants of 954 bp to − 945 bp (Mut1) and + 83 bp to + 94 bp (Mut2) sites for TNFα reporter and − 1973 bp to − 1964 bp (Mut4) site for IL-6 reporter (Supplementary Fig. S6B, D). These data support TNFα and IL-6 as the direct transcriptional targets of the RELA in largemouth bass, − 954 bp to − 945 bp and + 83 bp to + 94 bp in TNFα promoter regions, and − 1973 bp to − 1964 bp in IL-6 promoter regions were functional sites for RELA transactivation. Next, we sought to determine the impact of the acetylated and deacetylated RELA K119 on its transcriptional activity and DNA-binding. As indicated in Fig. 4B, C, RELA K119Q mutants led to approximately 50% reduction in the luciferase activity of TNFα and IL-6 reporter activity; a result in contrast to that of K119R compared to K119WT. Similarly, the Electrophoretic Mobility Shift Assay (EMSA) showed that K119R displayed a more enhanced DNA-binding affinity to the probe of TNFα promoter − 954 bp to − 945 bp compared with K119WT or K119Q (Fig. 4D). As RELA can shuttle between the nucleus and cytosol (Vermeulen et al. 2003), subsequently we examined whether the acetylation or deacetylation of RELA affects its subcellular localization in 293 T cells exogenously expressed RELA K119 WT and its mutants. Results from the immunofluorescence and western blot revealed that RELA K119R and K119Q preferentially located to the nucleus and cytosol, respectively (Fig. 4E–G).

To further investigate the function of RELA K119 mutants, we isolated the primary hepatocytes of largemouth bass and subjected them to adenovirus-mediated overexpression of RELA carrying either K119WT or mutant K119R. WT RELA or mutants were strongly expressed in primary hepatocytes of largemouth bass, as reflected by higher levels of RELA protein compared to the control virus (Supplementary Fig. S7A, B). Interestingly, the protein levels of TNFα, IL-1β and IL-8 were assessed by ELISA, and the mRNA levels of *TNFα, IL-6, IL-8, IL-1β, Mmp9, Bcl-2*, and *Bax* by qPCR were dramatically enhanced in primary hepatocytes overexpressing RELA K119R compared with those from RELA K119 WT under LPS stimulation (Fig. 4H, I). Together, these data supported that K119 deacetylation enhanced RELA DNA-binding affinity and transcriptional activity, suggesting that deacetylation of RELA K119 is critical to potentiate the transcription of pro-inflammatory genes in primary hepatocytes induced by LPS.

### RELA deacetylation is regulated by SIRT7

**Fig. 5:**
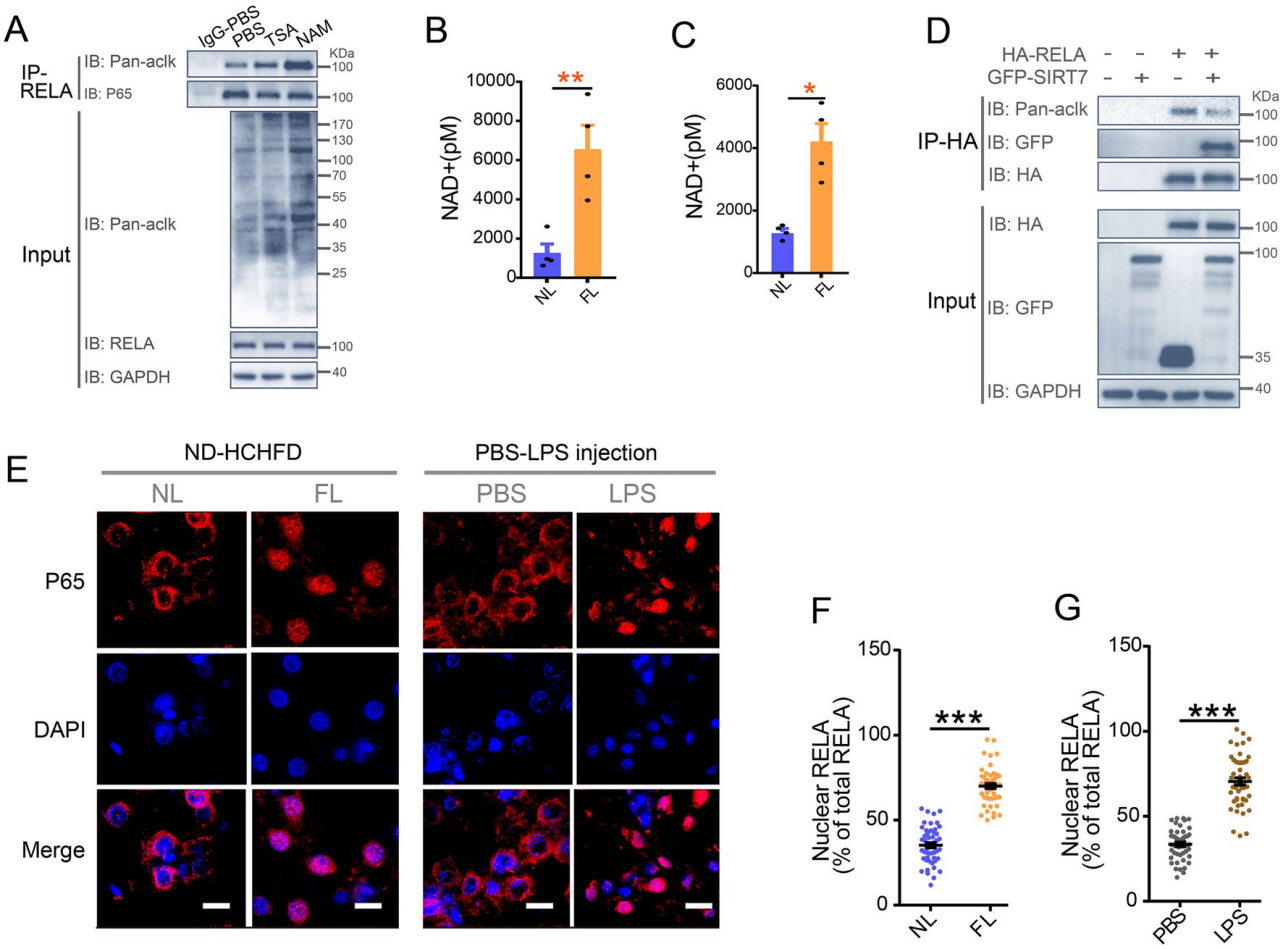
RELA physically interacts with SIRT7. **A** Fish were intraperitoneally injected with HDAC class I and II inhibitor Trichostatin A (TSA) (5 mg/kg), sirtuins inhibitor Nicotinamide (NAM) (500 mg/kg) or PBS control for 24 h prior to protein extraction for RELA immunoprecipitation followed by western blotting analysis of the acetylation of total protein and RELA. Representative western blot images for the indicated proteins (*n* = 4). B-C NAD + levels in normal livers (NL) and fibrotic livers (FL) from ND and HCHFD treated group (**B**) or Oil and CCL4 treated group (**C**) (*n* = 4). **D** Representative image of co-immunoprecipitation of RELA followed by western blot of the indicated proteins in HEK 293 T cells transfected with HA-tagged RELA and GFP-tagged SIRT7 plasmids as indicated for 24 h (*n* = 3). **E** Representative immunofluorescence images of RELA staining for the paraffin sections from normal liver (NL) and fibrotic liver (FL) induced with ND versus HCHFD, or from livers treated with PBS versus LPS (n = 4). Scale bar for E, 8 μm. **F**, **G** Quantitative results for panel E (*n* = 50 cells per group). Data were presented as mean ± SEM. *P*-values were determined by two-tailed unpaired Student’s *t*-tests

**Fig. 6:**
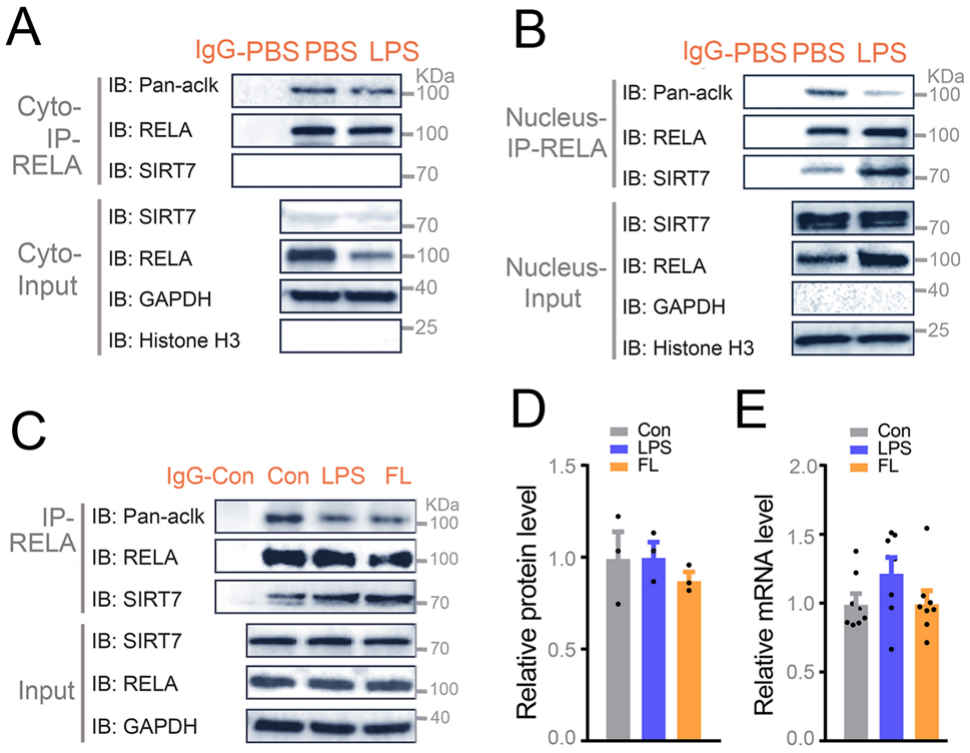
**A**, **B** Fish was intraperitoneally injected with LPS versus PBS for 24 h prior to cytosolic and nuclear protein extraction of fresh livers for co-immunoprecipitation of RELA followed by western blotting analysis of the acetylation of RELA and interaction between RELA and SIRT7 in cytosolic (**A**) and nucleus (**B**). Representative images were shown (*n* = 3). **C** Fresh livers from normal liver, fibrotic liver, and LPS-treated liver were used to extract protein for co-immunoprecipitation of RELA followed by western blotting analysis of the acetylation of RELA and interaction between RELA and SIRT7. Representative images for the indicated proteins (*n* = 3). **D** Quantitation results of endogenous SIRT7 protein expression in livers from panel J (*n* = 3). **E** qRT-PCR analysis of SIRT7 genes in normal liver, fibrotic liver, and LPS stimulated liver (*n* = 8). Data were presented as mean ± SEM. *P*-values were determined by one-way ANOVA corrected with Tukey’s multiple comparisons tests. *P < 0.05 was considered significant, **P < 0.01, ***P < 0.001

Deacetylation is mediated by histone deacetylase (HDAC) class I and II or sirtuins in mammals (Moreno-Yruela et al. 2022). To explore which deacetylase regulates the acetylation of RELA in largemouth bass, firstly we assessed the effect of HDAC class I and II inhibitor Trichostatin A (TSA) on protein acetylation in livers from fish, and primary hepatocytes. The acetylation of total protein and RELA were more significantly increased by NAM than TSA treatment in vivo and in vitro (Fig. 5A, Supplementary Fig. S9A) suggesting that deacetylation of RELA in largemouth bass is likely to be mediated by sirtuins. To test this likelihood, we assessed the contents of NAD + , an indispensable substrate for sirtuins, in normal versus HCHFD diet and CCL_4-_induced fibrotic livers. Thus, we found that the levels of NAD + were significantly enhanced in fibrotic livers (Fig. 5B, C), further supporting the notion that sirtuin family members (SIRT1-7) may play important roles as crucial regulators of liver fibrosis and the deacetylation of RELA. In addition, we determined that exogenously expressed RELA interacts with SIRT7, but not other sirtuin family members in HEK293T cells, including previously reported SIRT1 and SIRT2 in mammal (34, 40) by co-immunoprecipitation assay. Interestingly, the acetylation of RELA was downregulated by SIRT7 (Fig. 5D, Supplementary Fig. S10B). However, the acetylation of RELA (K119Q) was not affected by SIRT7 in HEK293T cells co-transfected with GFP-SIRT7 and HA-RELA (K119Q) suggesting that SIRT7 regulates the deacetylation of RELA is K119-dependent in largemouth bass. RELA resides in the cytoplasm in unstimulated cells. Upon cellular stress, it translocates to the nucleus (Chen et al. 2001). Next, we examined whether RELA and SIRT7 interact in hepatocytes. Thus, western blots of fractionated cytosolic and nuclear protein and immunofluorescence staining consistently showed that SIRT7 is mainly localized in the nucleus in liver of largemouth bass (Supplementary Fig. S8A, B), which is consistent with previously reported results in mammal (Mendes et al. 2017). Immunofluorescence showed RELA is predominantly located in the nucleus of hepatocytes of largemouth bass induced by HCHFD diet, CCL_4_ and LPS (Fig. 5E, F, G; Supplementary Fig. S9D, E). These results prompted us to test if RELA and SIRT7 interact in the nucleus of largemouth bass. Thus, we isolated cytoplasmic and nuclear protein extracts for co-IP assay, and found that the interaction between RELA and SIRT7 occurred only in the nuclear compartment and was significantly enhanced by LPS. Interestingly, the enhanced interaction between RELA and SIRT7 is accompanied by reduced levels of acetylated RELA protein. (Fig. 6A, B). Similar results were seen in fibrotic liver and LPS-treated acute inflamed liver (Fig. 6C). It was noteworthy that there were no obvious differences in the mRNA and protein levels of SIRT7 in normal, fibrotic and acute inflamed livers (Fig. 6D, E) suggesting that the increased interaction between RELA and SIRT7 was not caused by changes in SIRT7 protein level in liver. These results supported that SIRT7 interacts with RELA to promote the deacetylation of RELA in the nucleus in response to HCHFD and LPS stimulation.

### SIRT7 enhances the transcriptional activity of RELA

**Fig. 7:**
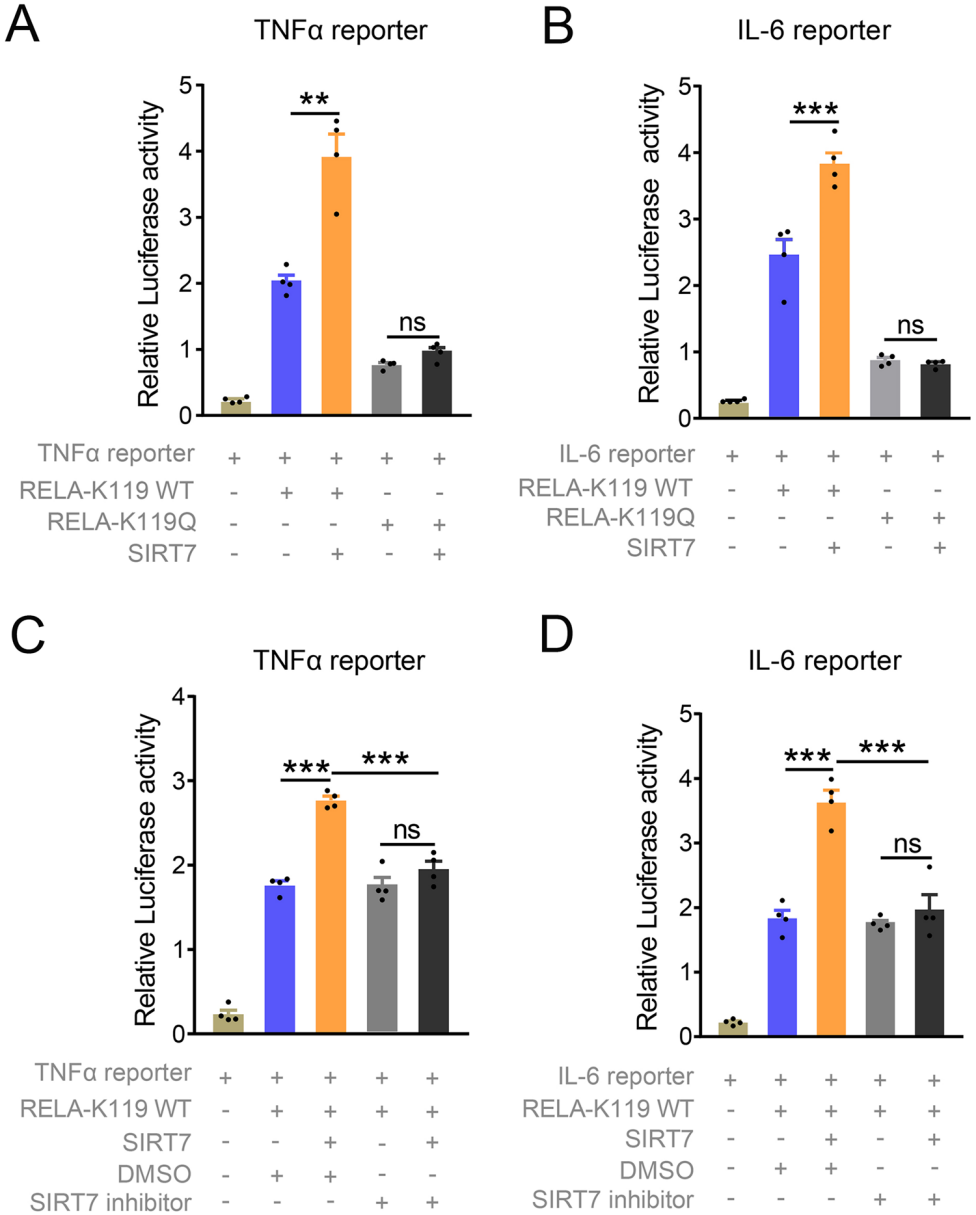
SIRT7 enhances TNFα and IL-6 promoter activity via inhibiting the acetylation of RELA-K119. **A**, **B** Luciferase assays for the effects of SIRT7 on TNFα (**A**) and IL-6 (**B**) promoter activity mediated by RELA K119WT and RELA K119Q in 293 T cells. Renilla reporter plasmid as an internal control. **C**, **D** Luciferase assays for TNFα (**C**) and IL-6 (**D**) promoter activity in HEK 293 T cells transfected with TNFα or IL-6 reporter plasmid and RELA K119WT and SIRT7 expression plasmid and exposed to SIRT7 inhibitor (10 µmol/L) or DMSO control for 24 h. Renilla reporter plasmid as an internal control. Data were presented as mean ± SEM (*n* = 3 independent experiments). *P*-values were determined by two-tailed unpaired Student’s t-tests. *P < 0.05 was considered significant, **P < 0.01, ***P < 0.001, ns, not significant

SIRT7 downregulates the acetylation of RELA in largemouth bass suggesting that SIRT7 may be involved in regulation of the RELA function. To test this hypothesis, we assessed the effects of SIRT7 on the transcriptional activity of RELA in HEK293T cells. In line with the findings in Fig. 4B and C, RELA K119 WT enhanced significantly the luciferase activity of TNFα and IL-6. Such an increase was further enhanced by overexpression of SIRT7. However, luciferase activity of TNFα mediated by RELA K119 Q was not affected by SIRT7 (Fig. 7A). Similar results were observed in luciferase activity of the IL-6 reporter (Fig. 7B). These results suggested that the regulation of SIRT7 on RELA transcriptional activity is K119-dependent in largemouth bass. To further investigate the effect of SIRT7 on the transcriptional activity of RELA, HEK293T cells were exposed to SIRT7 inhibitor (ID: 97,491) after co-transfection of SIRT7 and RELA K119 WT with TNFα or IL-6 reporter. Consistent with results in Fig. 7A and B, SIRT7 further strengthened RELA K119WT-activated TNFα and IL-6 Luciferase activity, but such increases was restored by inhibition of SIRT7 through a selective inhibitor (Fig. 7C, D). Of note, SIRT7 inhibitor did not affect the TNFα, and IL-6 luciferase activity mediated by RELA in the absence of SIRT7 expression. Taken together, these results suggest that SIRT7 enhances transcriptional activity of RELA by deacetylation of K119.

### Inhibition of SIRT7 reduces LPS-induced acute liver inflammation through attenuation of RELA deacetylation

**Fig. 8:**
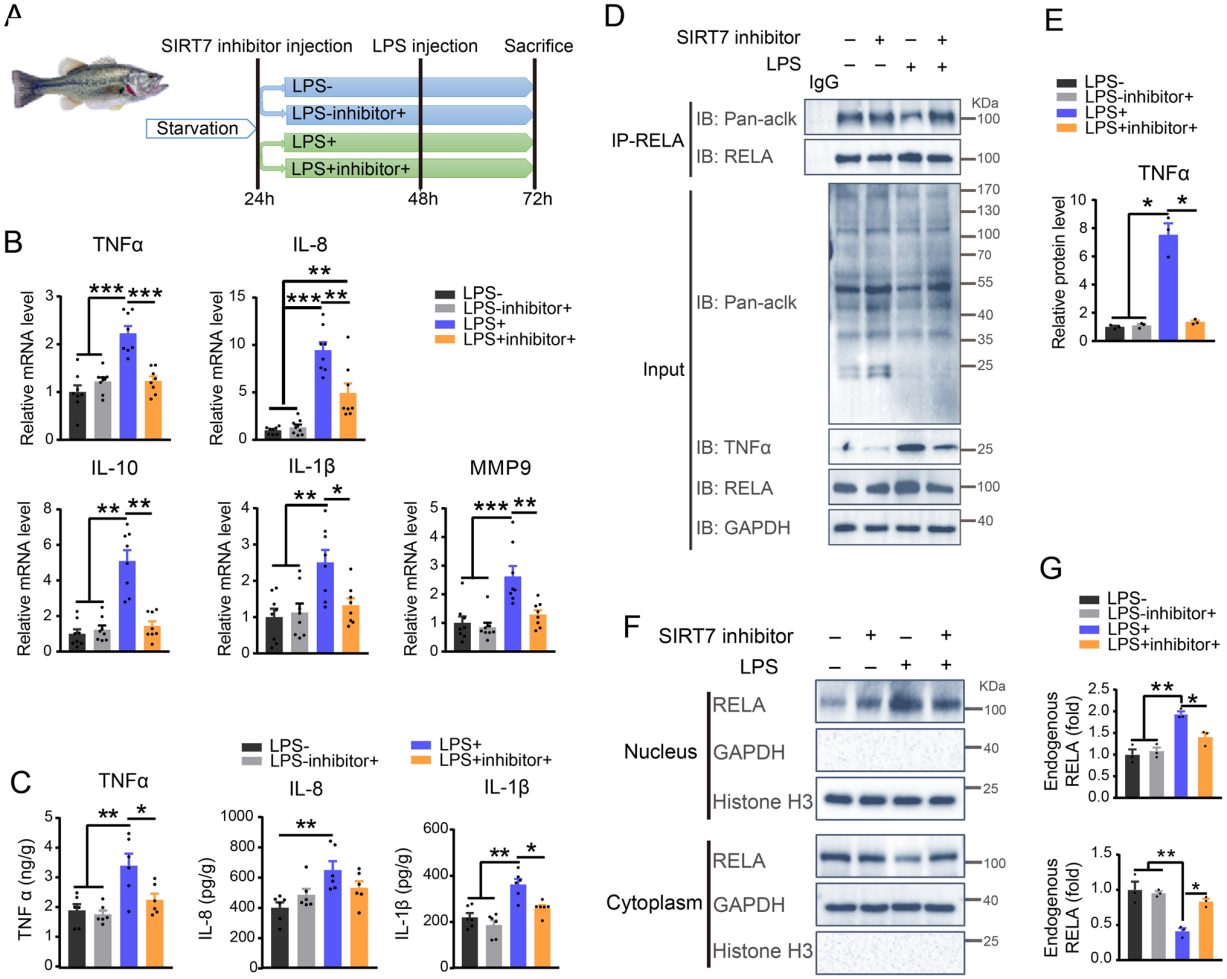
Inhibition of SIRT7 reduces LPS-induced inflammation in livers via reducing the deacetylation of RELA. **A** Schematic of experiment design. **B** qRT-PCR analysis of mRNA levels of the indicated proinflammatory genes in livers induced by LPS with or without SIRT7 inhibitor administration (*n* = 8). **C** Protein levels of TNFα, IL-1β, and IL-8 in the total protein extracted from liver assessed by ELISA (*n* = 8). **D** Representative western blot image for levels of TNFα, acetylated RELA and total protein in livers under the indicated conditions (*n* = 3). **E** Quantitation of the TNFα western blot in panel D (*n* = 3). **F** Representative western blot image for subcellular distribution of endogenous RELA in livers under the indicated conditions (*n* = 3). GAPDH and Histone H3 were included as positive controls for cytosolic and nuclear protein, respectively. **G** Quantitation of nucleus RELA(Top) and cytosolic RELA(Bottom) immunoblotting signals in panel F. Data were presented as mean ± SEM. *P*-values were determined by one-way ANOVA corrected with Tukey’s multiple comparisons tests in Fig. 8B (TNFα, IL-1β, MMP9) **C**, and **G** and corrected with Dunnett’s T3 post hoc tests in Fig. 8B (IL-8, IL-10) and **E**. *P < 0.05 was considered significant, **P < 0.01, ***P < 0.001

**Fig. 9:**
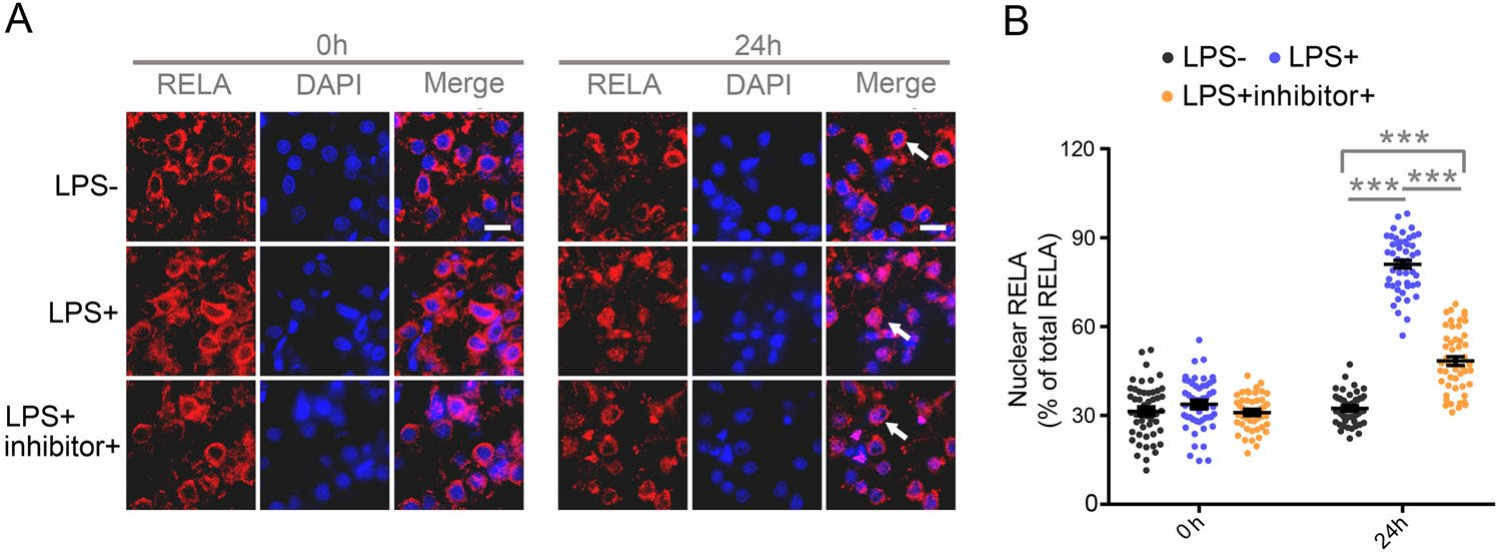
**A** Representative immunofluorescence images for RELA in the paraffin sections from the livers under the indicated conditions, nucleus was counterstained with DAPI (*n* = 4). White arrows denote signal location of RELA. Scale bar for A, 8 μm. **B** Quantitative results (*n* = 50 cells per group) in panel A. Data were presented as mean ± SEM. *P*-values were determined by one-way ANOVA corrected with Tukey’s multiple comparisons tests and corrected with Dunnett’s T3 post hoc tests.*P < 0.05 was considered significant, **P < 0.01, ***P < 0.001

To further determine the role of RELA deacetylation mediated by SIRT7 in largemouth bass, we established a LPS-induced fish model with acute liver inflammation and isolated primary hepatocytes followed by LPS stimulation (Fig. 8A, Supplementary Fig. S10A). The largemouth bass that received LPS stimulation showed increased mRNA levels of RELA target genes related to inflammation, such as TNFα, IL-8, IL-10, IL-1β, Mmp9, and the protein level of TNFα, IL-8, IL-1β in liver. Such an increase was abolished by SIRT7 inhibitor treatment (Fig. 8B, C). Consistent results were observed in primary hepatocytes (Supplementary Fig. S10B, C). Next, we examined whether the effect of inhibiting LPS-mediated inflammation in vivo and in vitro by SIRT7 inhibition was relevant to the deacetylation of RELA. Notably, the acetylation of total protein and RELA was markedly decreased, whereas TNFα expression was dramatically elevated in LPS-stimulated liver tissue and primary hepatocytes (Fig. 8 D, E, Supplementary Fig. S10D), and such reduction was rescued by SIRT7 inhibition. Together, these results suggested that inhibition of SIRT7 attenuated LPS-induced liver inflammation was at least in part through suppression of the deacetylation of RELA. Because acetylation of RELA affects its cellular localization, we examined whether SIRT7 inhibition affects the subcellular distribution of RELA in livers after LPS stimulation. The results showed that the protein level of RELA was increased considerably in nuclei following LPS stimulation, which was suppressed by SIRT7 inhibitor in vivo (Fig. 8F, G) and in vitro (Supplementary Fig. S10F, G). Of note, cytoplasmic localization of RELA was opposite to nuclear localization. Similarly, immunofluorescence staining illustrated that most of RELA was located mainly in nuclei after 24 h of LPS stimulation, with SIRT7 inhibitor decreasing such nuclear distribution of RELA in vivo (Fig. 9A, B). Collectively, these data suggested that SIRT7-dependent RELA deacetylation ameliorates liver inflammation and prevents nuclear localization of RELA.

### Inhibition of SIRT7 ameliorates fibrogenesis in livers of largemouth bass

**Fig. 10:**
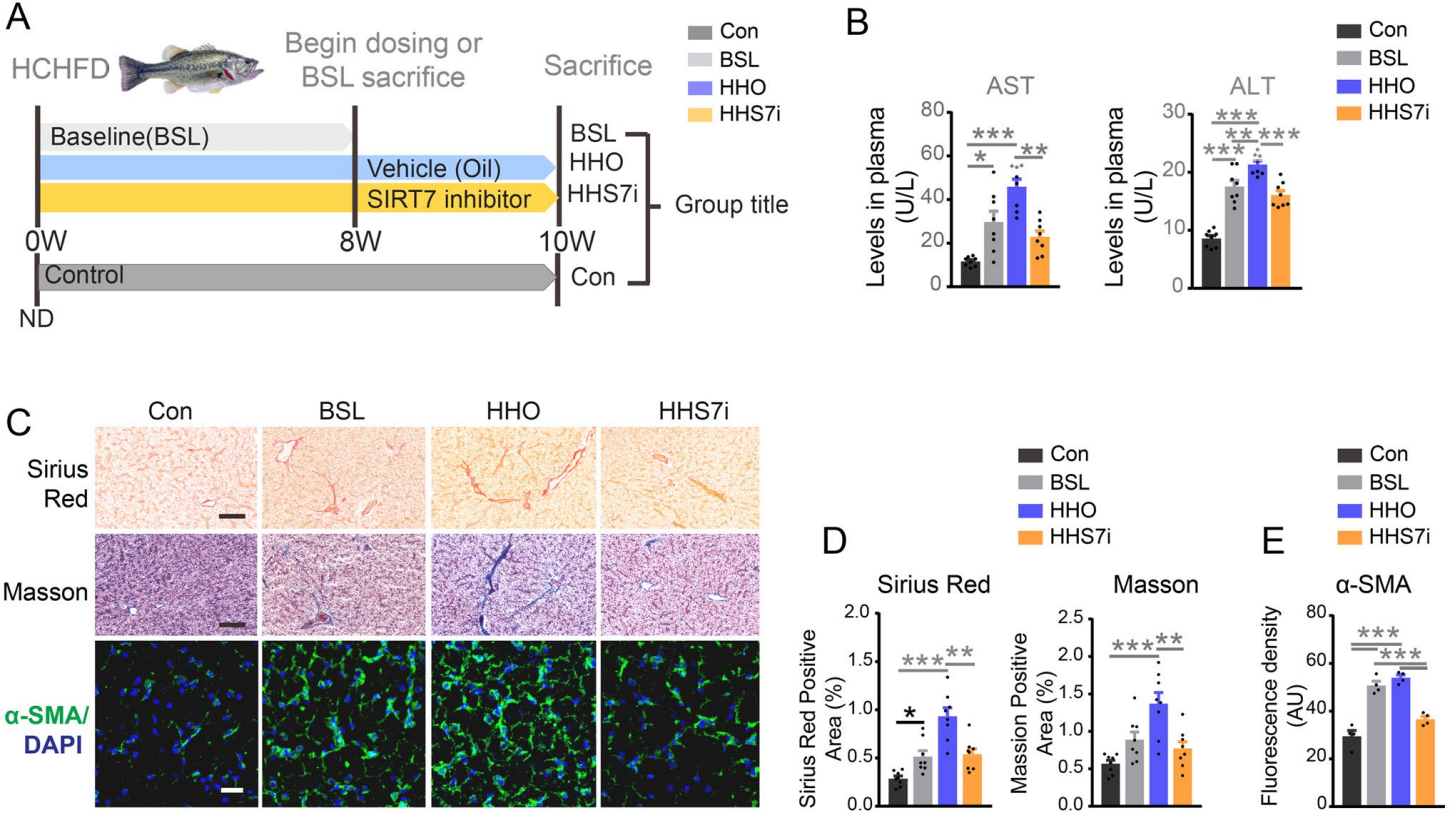
Inhibition of SIRT7 ameliorates fibrosis in the livers induced by long-term HCHFD. **A** Schematic of experiment design. **B** Protein levels of AST and ALT in plasma from indicated groups were detected by ELISA (*n* = 8). **C**–**E** Representative images of Sirius Red and Masson staining (*n* = 8), and representative immunofluorescence images of α-SMA (*n* = 4) for paraffin sections from livers of indicated groups (**C**). Black scale bar represents 50 µm, White scale bar represents 20 µm for **C**. Quantitation for Sirius Red (Right) and Masson staining (Left) (**D**) (*n* = 8), and for fluorescence density of α-SMA (**E**) (*n* = 4) in panel C. Data were presented as mean ± SEM. *P*-values were determined by one-way ANOVA corrected with Tukey’s multiple comparisons test in Fig. 10 B (ALT), **D** and **E** and corrected with Dunnett’s T3 post hoc test in Fig. 10 B (AST). *P < 0.05 was considered significant, **P < 0.01, ***P < 0.001

**Fig. 11:**
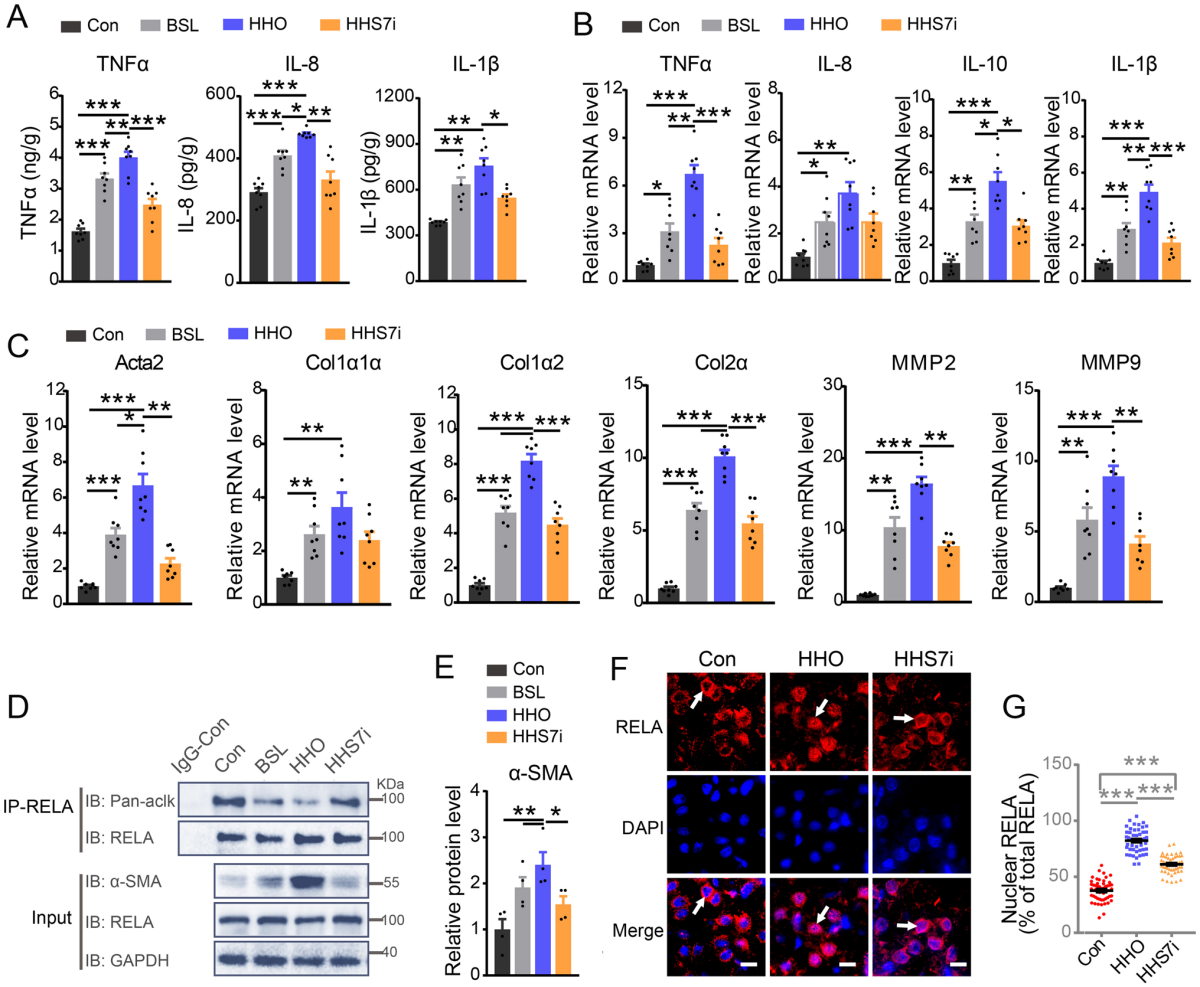
**A** Protein levels of the indicated proinflammatory factors in the total protein from the livers under the indicated conditions were measured by ELISA (*n* = 8). **B**, **C** qRT-PCR analysis of the indicated inflammation related genes (**B**) and fibrosis related genes (**C**) in livers under the indicated conditions (*n* = 8). **D**, **E** Representative western blot images for indicated proteins in the livers under the indicated conditions (**D**) and the quantitation of α-SMA protein level (**E**) (*n* = 4). **F** Representative immunofluorescence images of endogenous RELA in the livers of indicated groups (*n* = 4). Scale bar for F, 8 μm. **G** Quantitative results for panel F (*n* = 50 cells per group). Data were presented as mean ± SEM. *P*-values were determined by one-way ANOVA corrected with Tukey’s multiple comparisons test in Fig. 11A (TNFα), **E** and **G** and corrected with Dunnett’s T3 post hoc test in Fig. 11 A (IL-8, IL-1β), B and C. *P < 0.05 was considered significant, **P < 0.01, ***P < 0.001

Given the potent effects of SIRT7 inhibitor on suppression of LPS-mediated liver inflammation, we examined whether SIRT7 inhibitor could exert a similar role in suppressing liver fibrosis in a fish model induced by chronic inflammation preventing RELA deacetylation. Fish were fed HCHFD for eight weeks, followed by SIRT7 inhibitor intervention for a further two weeks. Disease baseline was determined at eight weeks such that comparisons were made between baseline and other groups at the end of the study (week 10) representing disease progression (Fig. 10A). Compared to the controls (Con), the baseline (BSL) group exhibited an elevation of serum ALT and AST contents (Fig. 10B) and collagenous fiber accumulation (Fig. 10C–E). The mRNA levels of proinflammatory genes, including *TNFα, IL-8, IL-10* and *IL-1β* (Fig. 11B), and molecular signatures of fibrosis important, such as *Acta2, Col1α1α, Col1α2, Col2α*, *Mmp2* and *Mmp9* (Fig. 11C) were enhanced markedly in the BSL group. Furthermore, western blots and ELISA showed that the protein levels of α-SMA and inflammatory factors TNFα, IL-8 and IL-1β were increased significantly in BSL compared to the controls (Fig. 11A, D, E). Furthermore, all the above parameters were elevated in the HHO (HCHFD with oil administration) group indicating that fish had pre-established liver fibrosis with inflammation at the initiation of treatment in this study. Compared to the HHO group, elevated collagen deposition and serum ALT and AST contents were blunted by SIRT7 inhibitor intervention (Fig. 10B–D). Significantly elevated mRNA levels of inflammation related genes, such as *TNFα, IL-8* and *IL-1β*, and molecular signatures of fibrosis, such as *Acta2, Col1α1α, Col1α2, Col2α*, *Mmp2* and *Mmp9* in the HHO group were decreased to some extent in the HHS7i group (HCHFD with SIRT7 inhibition) (Fig. 11B, C). Similar results were observed in secretion of TNFα, IL-8, and IL-1β in livers by ELISA (Fig. 11A), and in α-SMA expression by western blot and immunofluorescence analysis (Fig. 10C, E, Fig. 11D, E). Taken together, these data indicated that inhibition of SIRT7 eliminates liver inflammation and fibrosis induced by HCHFD.

**Fig. 12:**
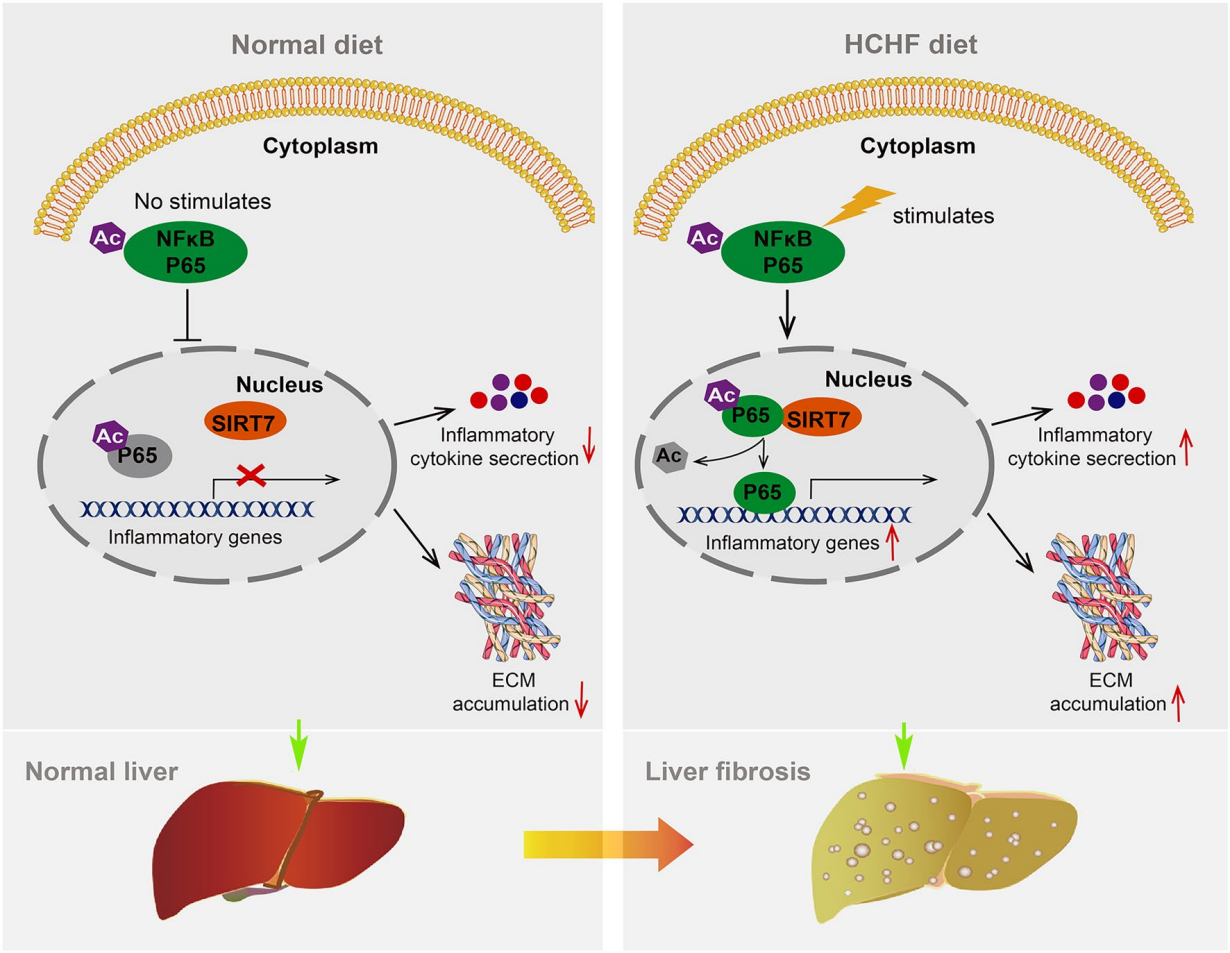
Schematic of working model: RELA shuttles from cytoplasm into the nucleus under the stress stimulation and then interacts with SIRT7, resulting in RELA K119 deacetylation and activation. This promotes the expression of the proinflammatory genes to aggravate the development of liver fibrosis in largemouth bass

Subsequently, we examined whether the favorable effects on mitigating liver fibrosis and liver inflammation mediated by SIRT7 inhibition is through its influence on the acetylation of RELA. As shown in Fig. 11D, the acetylation of RELA declined remarkably in the BSL and HHO groups, with aggravated liver inflammation and fibrosis compared to the control. However, such reduction was reversed by SIRT7 inhibition. Similar to results in Fig. 8 F, G and Fig. 9 A, B, long-term HCHFD treatment stimulated RELA translocation from cytoplasm to nucleus. However, the nuclear RELA was decreased under the intervention of SIRT7 inhibitor (Fig. 11F, G). Of note, over-inhibition of RELA leads to cell apoptosis and accelerates liver injury (Iimuro et al. 1998). Although inhibition of SIRT7 significantly reduces the expression of RELA target genes in liver fibrosis induced by HCHFD, it has no effect on overall liver cell apoptosis (Supplementary Fig. S11). These data support that inhibition of SIRT7 prevents the downregulation of RELA acetylation, and thus prevents fibrogenesis in livers of largemouth bass induced by HCHFD suggesting that the SIRT7-RELA regulatory axis may represent a potential target for prevention and treatment of liver fibrosis in fish (Fig. 12).

## Discussion

In the present study, we demonstrate that total protein and RELA acetylation are significantly decreased in liver fibrosis and acute liver inflammation in vivo, and in primary hepatocytes stimulated by LPS. Our findings shed light on a new mechanism as to how RELA deacetylation of K119 enhances liver inflammation and fibrosis via SIRT7 in largemouth bass. We propose a model in which stress promotes RELA nuclear translocation to facilitate the interaction with SIRT7, leading to enhanced RELA deacetylation and the subsequent aggravation of liver inflammation and the development of liver fibrosis. Also, our studies indicate that reconstitution of adequate RELA acetylation by inhibiting SIRT7 ameliorates liver inflammation and fibrosis (Fig. 8).

### Post-translational modifications of RELA represent a promising therapeutic avenue for liver fibrosis and inflammation

Liver fibrosis is the result of chronic liver injury of different etiologies, and usually may be reversed after cessation of injury (Hammel et al. 2001). Uncontrolled inflammation has been regarded as a vital driving force in transforming self-limited tissue repair processes into a vicious loop, and accelerating the development of liver fibrosis (Pellicoro et al. 2014). In the present study, dramatically elevated inflammation was found in fibrotic liver induced by HCHFD and CCL_4_, implying that limiting inflammation may represent a prospective approach to prevent deterioration of liver fibrosis in largemouth bass. NFκB is a widely recognized transcription factor involved in the inflammatory response, and has been recognized as a target to manage tissue inflammation and the development of liver fibrosis (Luedde and Schwabe 2011). However, NFκB acts as a double-edged sword, inhibiting NFκB activity may not only exert antifibrogenic effects by downregulation of inflammation (Wang et al. 2020) or directly reduction of the transcription of the Col1α1 gene (Rippe et al. 1999), but also exert profibrogenic effects by increasing hepatocytes injury, especially when NFκB inhibition is pronounced (Luedde and Schwabe 2011). Accordingly, excessive activation of NFκB results in aggravated liver fibrosis by triggering secretion of inflammatory and chemotactic factors (Higashi et al. 2017) or activating TGFβ signaling to sensitize quiescent HSCs (Seki et al. 2007). The dual role of NFκB demands a delicate balance given that over-activation or over-inhibition of NFκB may both have an adverse impact on the liver fibrosis. There is probably a threshold through which NFκB inhibition prevents the secretion of excessive inflammatory factors and aggravation of liver inflammation and fibrosis, which necessitates cell homeostasis. However, no such threshold has yet been precisely determined. Therefore, a new solution should be explored. Based on the potent effect of PTMs on the function of NFκB, manipulating the function of NFκB via targeting PTMs may represent a promising approach to control liver fibrosis and inflammation. Our data showed that inhibiting RELA, a subunit of NFκB, activity through increasing its acetylation exerts a beneficial role of blunting liver inflammation and fibrosis by suppressing the secretion of inflammatory factors and the expression of profibrogenic genes. However, it does not lead to mass death of hepatocytes (Supplementary Fig. S13). We argue that transformation of NFκB biology to novel therapies is more likely to emerge from a re-focus of basic research back to functional regulation of NFκB subunit through PTMs (Chakraborty and Mann 2010).

### Deacetylation of RELA K119 is a site- and species-specific mechanism driving liver fibrosis pathogenesis in Largemouth bass

At present, how the acetylation of RELA regulates the pathogenesis of liver fibrosis is still unknown, even in mammals. Our data provided evidence that deacetylated RELA is a central link for the aberrant elevation of liver inflammation and fibrosis in largemouth bass. RELA K119 deacetylation promotes its DNA-binding activity and enhances the expression of target genes, supporting the idea that deacetylation of RELA is responsible for inflammation response to liver injury. Conservation analyses indicated that K119 of largemouth bass and K105 of homo sapiens lie in the Rel homology domain (RHD), and is a conserved DNA-binding site. However, no studies have yet reported on the acylation and function of the K105 site of human RELA protein. Previous studies revealed that acetylation of K310, K314 and K315 is necessary for regulating the specificity of RELA-dependent gene expression (Buerki et al. 2008; Rothgiesser et al. 2010b). Acetylation of K218 and K221 enhanced DNA-binding activity of RELA to increase its response in the nucleus, whreas acetylation of K122 and K123 decreased DNA-binding of RELA to terminate the RELA response (Chen et al. 2001, 2002; Furia et al. 2002; Kiernan et al. 2003). Together these findings suggest that acetylation of RELA regulation of its functional characteristics is site-and species-dependent. Our study provides novel insights into how the acetylation at different lysine sites influences RELA functions, which may have new implications on effective strategies for fighting against liver inflammation and fibrosis.

### SIRT7 specifically mediates the deacetylation of RELA in largemouth bass

Deacetylation of RELA is regulated by sirtuins (SIRT1, SIRT2) and HDACs (HDAC1, HDAC3) in mice and humans, and exerts different effects on its function (Chen et al. 2018; Qin et al. 2017; Quivy and Van Lint 2004; Yeung et al. 2004). In our study, NAM, an inhibitor of the sirtuin family, had a stronger impact on acetylation of RELA than TSA, an inhibitor of HDACs (Fig. 5 A, Supplementary Fig. S10A). This indicates that sirtuins may serve as the primary deacetylase in largemouth bass. Furthermore, SIRT7, but not other sirtuins, interacted with RELA to deacetylate K119 resulting in enhanced RELA transcription activity and expression of target genes. SIRT7 is a nuclear sirtuin family member possessing deacetylase, desuccinylase and deacylase activities (Ford et al. 2006; Li et al. 2016, 2022; Tong et al. 2017). Consistent with our study, SIRT7 is located mainly in the nucleus (Supplementary Fig. S9A, B), suggesting that deacetylation of RELA in a stress response is dependent on nuclear SIRT7. RELA primarily locates in the cytoplasm under physiologic circumstances, but it translocated to the nucleus under HCHFD, CCL_4_ challenge, or LPS stimulation in largemouth bass (Fig. 5E, Fig. 9A, Supplementary Fig. S10D). However, whether the interaction between RELA and SIRT7 occurs only in the nucleus remains unknown. Our results demonstrated a clear nuclear interaction of RELA and SIRT7, which was potentiated by a lower acetylation of nuclear RELA under LPS treatment. In line with this, we also found that the acetylation of RELA was dramatically decreased, accompanied by a significant elevation in the interaction between SIRT7 and RELA and in expression of target genes in HCHFD or CCL_4_-induced fibrotic liver and LPS-treated liver (Fig. 6A–C). The above results indicated that the influence of SIRT7 on RELA deacetylation requires nuclear interaction of these two molecules. Interestingly, after inhibition of SIRT7 by a novel inhibitor of SIRT7 (ID: 97,491), the decreased acetylation of RELA was restored, leading to an evident remission of liver inflammation and fibrosis under HCHFD and LPS treatment (Figs. 8, 10, 11). Previous studies demonstrated that inhibition of SIRT7 greatly enhanced LPS-stimulated inflammatory mediator secretion by NFκB signaling pathway independent of acetylation in dairy cow mammary epithelial cells (DCMECs) (Chen et al. 2019), but the mechanism is unclear. Thus, the regulation of RELA acetylation and its functional characteristics by SIRT7 is species- specific. Our study has revealed a previously unrecognized molecular link between the deacetylation of RELA and its transcription activity, and expression of target genes by SIRT7, which further regulates the liver inflammation and the development of liver fibrosis in largemouth bass. The findings in this study provide deeper insights into the role of acetylation of RELA in regulating liver inflammation and fibrosis, which improves our knowledge on the prevention of liver fibrosis in humans from the perspective of PTMs. Nonetheless, additional research is necessary to clarify in-depth molecular mechanisms.

In conclusion, this study reports that SIRT7 deacetylates RELA K119 in the nucleus, consequently increasing the transactivation of RELA in the proinflammatory gene program. This in turn elevates the release of inflammatory factors and worsens liver fibrosis in largemouth bass. These findings improved our understanding of PTMs regulation of liver inflammation and fibrosis, and provided a potential target for liver fibrosis therapy and genomic breeding for largemouth bass and other carnivorous fish.

## Supplementary Information

Below is the link to the electronic supplementary material.

Supplementary file1 (XLSX 35 KB)

Supplementary file2 (DOC 8490 KB)

## Data Availability

All data supporting the main findings of this study are available within the article and its Supplementary Information files or from the corresponding author upon reasonable request.

## References

[CR1] Alegre F, Pelegrin P, Feldstein AE (2017) Inflammasomes in liver fibrosis. Semin Liver Dis 37:119–127 28564720 10.1055/s-0037-1601350

[CR2] Biessels GJ, Luchsinger JA (2010) Diabetes and the brain. Humana Press, New York

[CR3] Buerki C, Rothgiesser KM, Valovka T, Owen HR, Rehrauer H, Fey M, Lane WS, Hottiger MO (2008) Functional relevance of novel p300-mediated lysine 314 and 315 acetylation of RelA/p65. Nucleic Acids Res 36:1665–1680 18263619 10.1093/nar/gkn003 PMC2275151

[CR4] Chakraborty JB, Mann DA (2010) NF-kappaB signalling: embracing complexity to achieve translation. J Hepatol 52:285–291 20022129 10.1016/j.jhep.2009.10.030

[CR5] Chen L, Fischle W, Verdin E, Greene WC (2001) Duration of nuclear NF-kappaB action regulated by reversible acetylation. Science 293:1653–1657 11533489 10.1126/science.1062374

[CR6] Chen LF, Mu Y, Greene WC (2002) Acetylation of RelA at discrete sites regulates distinct nuclear functions of NF-kappaB. EMBO J 21:6539–6548 12456660 10.1093/emboj/cdf660 PMC136963

[CR7] Chen LF, Williams SA, Mu Y, Nakano H, Duerr JM, Buckbinder L, Greene WC (2005) NF-kappaB RelA phosphorylation regulates RelA acetylation. Mol Cell Biol 25:7966–7975 16135789 10.1128/MCB.25.18.7966-7975.2005 PMC1234328

[CR8] Chen S, Ye J, Chen X, Shi J, Wu W, Lin W, Lin W, Li Y, Fu H, Li S (2018) Valproic acid attenuates traumatic spinal cord injury-induced inflammation via STAT1 and NF-kappaB pathway dependent of HDAC3. J Neuroinflamm 15:150 10.1186/s12974-018-1193-6 PMC5960086 29776446

[CR9] Chen KL, Li L, Li CM, Wang YR, Yang FX, Kuang MQ, Wang GL (2019) SIRT7 regulates lipopolysaccharide-induced inflammatory injury by suppressing the NF-kappaB signaling pathway. Oxid Med Cell Longev 2019:3187972 31285783 10.1155/2019/3187972 PMC6594283

[CR10] Chen P, Wu X, Gu X, Han J, Xue M, Liang X (2021a) FoxO1 in *Micropterus salmoides* : molecular characterization and its roles in glucose metabolism by glucose or insulin-glucose loading. Gen Comp Endocrinol 310:113811 33979571 10.1016/j.ygcen.2021.113811

[CR11] Chen Q, Du J, Cui K, Fang W, Zhao Z, Chen Q, Mai K, Ai Q (2021b) Acetyl-CoA derived from hepatic mitochondrial fatty acid beta-oxidation aggravates inflammation by enhancing p65 acetylation. iScience 24:103244 34746707 10.1016/j.isci.2021.103244 PMC8551082

[CR12] Ford E, Voit R, Liszt G, Magin C, Grummt I, Guarente L (2006) Mammalian Sir2 homolog SIRT7 is an activator of RNA polymerase I transcription. Genes Dev 20:1075–1080 16618798 10.1101/gad.1399706 PMC1472467

[CR13] Furia B, Deng L, Wu K, Baylor S, Kehn K, Li H, Donnelly R, Coleman T, Kashanchi F (2002) Enhancement of nuclear factor-kappa B acetylation by coactivator p300 and HIV-1 Tat proteins. J Biol Chem 277:4973–4980 11739381 10.1074/jbc.M107848200

[CR14] Gong YL, Lu QS, Liu YL, Xi LW, Zhang ZM, Liu HK, Jin JY, Yang YX, Zhu XM, Xie SQ, Han D (2022) Dietary berberine alleviates high carbohydrate diet-induced intestinal damages and improves lipid metabolism in largemouth bass (*Micropterus salmoides*). Front Nutr 9:1010859 36211485 10.3389/fnut.2022.1010859 PMC9539808

[CR15] Hammel P, Couvelard A, O’Toole D, Ratouis A, Sauvanet A, Flejou JF, Degott C, Belghiti J, Bernades P, Valla D, Ruszniewski P, Levy P (2001) Regression of liver fibrosis after biliary drainage in patients with chronic pancreatitis and stenosis of the common bile duct. N Engl J Med 344:418–423 11172178 10.1056/NEJM200102083440604

[CR16] Hernandez-Gea V, Friedman SL (2011) Pathogenesis ofliver fibrosis. Annu Rev Pathol-Mech 6:425–456 10.1146/annurev-pathol-011110-130246 21073339

[CR17] Higashi T, Friedman SL, Hoshida Y (2017) Hepatic stellate cells as key target in liver fibrosis. Adv Drug Deliv Rev 121:27–42 28506744 10.1016/j.addr.2017.05.007 PMC5682243

[CR18] Hung CT, Su TH, Chen YT, Wu YF, Chen YT, Lin SJ, Lin SL, Yang KC (2022) Targeting ER protein TXNDC5 in hepatic stellate cell mitigates liver fibrosis by repressing non-canonical TGFβ signaling. Gut 71:1876–1891 34933915 10.1136/gutjnl-2021-325065

[CR19] Iimuro Y, Nishiura T, Hellerbrand C, Behrns KE, Schoonhoven R, Grisham JW, Brenner DA (1998) NFkappaB prevents apoptosis and liver dysfunction during liver regeneration. J Clin Invest 101:802–811 9466975 10.1172/JCI483 PMC508628

[CR20] Karve TM, Cheema AK (2011) Small changes huge impact: the role of protein posttranslational modifications in cellular homeostasis and disease. J Amino Acids 2011:207691 22312457 10.4061/2011/207691 PMC3268018

[CR21] Kiernan R, Bres V, Ng RW, Coudart MP, El Messaoudi S, Sardet C, Jin DY, Emiliani S, Benkirane M (2003) Post-activation turn-off of NF-kappa B-dependent transcription is regulated by acetylation of p65. J Biol Chem 278:2758–2766 12419806 10.1074/jbc.M209572200

[CR22] Kohli R, Kirby M, Xanthakos SA, Softic S, Feldstein AE, Saxena V, Tang PH, Miles L, Miles MV, Balistreri WF, Woods SC, Seeley RJ (2010) High-Fructose, medium chain trans fat diet induces liver fibrosis and elevates plasma coenzyme Q9 in a novel murine model of obesity and nonalcoholic steatohepatitis. Hepatology 52:934–944 20607689 10.1002/hep.23797 PMC2932817

[CR23] Koyama Y, Brenner DA (2017) Liver inflammation and fibrosis. J Clin Invest 127:55–64 28045404 10.1172/JCI88881 PMC5199698

[CR24] Li L, Shi L, Yang S, Yan R, Zhang D, Yang J, He L, Li W, Yi X, Sun L, Liang J, Cheng Z, Shi L, Shang Y, Yu W (2016) SIRT7 is a histone desuccinylase that functionally links to chromatin compaction and genome stability. Nat Commun 7:12235 27436229 10.1038/ncomms12235 PMC4961794

[CR25] Li S, Monroig O, Wang T, Yuan Y, Carlos Navarro J, Hontoria F, Liao K, Tocher DR, Mai K, Xu W, Ai Q (2017) Functional characterization and differential nutritional regulation of putative Elovl5 and Elovl4 elongases in large yellow croaker (*Larimichthys crocea*). Sci Rep 7:2303 28536436 10.1038/s41598-017-02646-8 PMC5442133

[CR26] Li X, Zheng S, Ma X, Cheng K, Wu G (2020) Effects of dietary starch and lipid levels on the protein retention and growth of largemouth bass (*Micropterus salmoides*). Amino Acids 52:999–1016 32648068 10.1007/s00726-020-02869-6

[CR27] Li M, Xiong J, Yang L, Huang J, Zhang Y, Liu M, Wang L, Ji J, Zhao Y, Zhu WG, Luo J, Wang H (2022) Acetylation of p62 regulates base excision repair through interaction with APE1. Cell Rep 40:111116 35858573 10.1016/j.celrep.2022.111116

[CR28] Liu R, Zhong Y, Li X, Chen H, Jim B, Zhou MM, Chuang PY, He JC (2014) Role of transcription factor acetylation in diabetic kidney disease. Diabetes 63:2440–2453 24608443 10.2337/db13-1810 PMC4066331

[CR29] Liu T, Zhang L, Joo D, Sun SC (2017) NF-kappaB signaling in inflammation. Signal Transduct Target Ther 2:17023 29158945 10.1038/sigtrans.2017.23 PMC5661633

[CR30] Liu Y, Nong L, Jia YX, Tan AH, Duan LX, Lu YK, Zhao JM (2020) Aspirin alleviates hepatic fibrosis by suppressing hepatic stellate cells activation via the TLR4/NF-κB pathway. Aging-Us 12:6058–6066 10.18632/aging.103002 PMC7185140 32283542

[CR31] Liu YJ, Liu N, Wang A, Chen NS, Li SL (2022) Resveratrol inclusion alleviated high-dietary-carbohydrate-induced glycogen deposition and immune response of largemouth bass, *Micropterus salmoides*. Brit J Nutr 127:165–176 33583445 10.1017/S0007114521000544

[CR32] Lu J, Huang X, Deng A, Yao H, Wu G, Wang N, Gui H, Ren M, Guo S (2022) miR-452-3p targets HDAC3 to inhibit p65 deacetylation and activate the NF-kappaB signaling pathway in early brain injury after subarachnoid hemorrhage. Neurocrit Care 37:558–571 35641805 10.1007/s12028-022-01509-z

[CR33] Luedde T, Schwabe RF (2011) NF-kappaB in the liver–linking injury, fibrosis and hepatocellular carcinoma. Nat Rev Gastroenterol Hepatol 8:108–118 21293511 10.1038/nrgastro.2010.213 PMC3295539

[CR34] Ma Z, Chalkley RJ, Vosseller K (2017) Hyper-O-GlcNAcylation activates nuclear factor kappa-light-chain-enhancer of activated B cells (NF-kappaB) signaling through interplay with phosphorylation and acetylation. J Biol Chem 292:9150–9163 28416608 10.1074/jbc.M116.766568 PMC5454098

[CR35] Mendes KL, Lelis DF, Santos SHS (2017) Nuclear sirtuins and inflammatory signaling pathways. Cytokine Growth Factor Rev 38:98–105 29132743 10.1016/j.cytogfr.2017.11.001

[CR36] Moreno-Yruela C, Zhang D, Wei W, Baek M, Liu W, Gao J, Dankova D, Nielsen AL, Bolding JE, Yang L, Jameson ST, Wong J, Olsen CA, Zhao Y (2022) Class I histone deacetylases (HDAC1–3) are histone lysine delactylases. Sci Adv 8:eabi6696 35044827 10.1126/sciadv.abi6696 PMC8769552

[CR37] Pellicoro A, Ramachandran P, Iredale JP, Fallowfield JA (2014) Liver fibrosis and repair: immune regulation of wound healing in a solid organ. Nat Rev Immunol 14:181–194 24566915 10.1038/nri3623

[CR38] Qin K, Han C, Zhang H, Li T, Li N, Cao X (2017) NAD(+) dependent deacetylase Sirtuin 5 rescues the innate inflammatory response of endotoxin tolerant macrophages by promoting acetylation of p65. J Autoimmun 8:120–129 10.1016/j.jaut.2017.04.006 28461090

[CR39] Quivy V, Van Lint C (2004) Regulation at multiple levels of NF-kappaB-mediated transactivation by protein acetylation. Biochem Pharmacol 68:1221–1229 15313420 10.1016/j.bcp.2004.05.039

[CR40] Rippe RA, Schrum LW, Stefanovic B, Solis-Herruzo JA, Brenner DA (1999) NF-kappaB inhibits expression of the alpha1(I) collagen gene. DNA Cell Biol 18:751–761 10541434 10.1089/104454999314890

[CR41] Rothgiesser KM, Erener S, Waibel S, Luscher B, Hottiger MO (2010a) SIRT2 regulates NF-kappaB-dependent gene expression through deacetylation of p65 Lys310. J Cell Sci 123:4251–4258 21081649 10.1242/jcs.073783

[CR42] Rothgiesser KM, Fey M, Hottiger MO (2010b) Acetylation of p65 at lysine 314 is important for late NF-kappaB-dependent gene expression. BMC Genomics 11:22 20064247 10.1186/1471-2164-11-22 PMC2823688

[CR43] Sato Y, Hashiguchi Y, Nishida M (2009) Temporal pattern of loss/persistence of duplicate genes involved in signal transduction and metabolic pathways after teleost-specific genome duplication. BMC Evol Biol 9:127 19500364 10.1186/1471-2148-9-127 PMC2702319

[CR44] Seki E, De Minicis S, Osterreicher CH, Kluwe J, Osawa Y, Brenner DA, Schwabe RF (2007) TLR4 enhances TGF-beta signaling and hepatic fibrosis. Nat Med 13:1324–1332 17952090 10.1038/nm1663

[CR45] Tong Z, Wang M, Wang Y, Kim DD, Grenier JK, Cao J, Sadhukhan S, Hao Q, Lin H (2017) SIRT7 is an RNA-activated protein lysine deacylase. ACS Chem Biol 12:300–310 27997115 10.1021/acschembio.6b00954 PMC5326686

[CR46] Tsuchida T, Friedman SL (2017) Mechanisms of hepatic stellate cell activation. Nat Rev Gastro Hepat 14:397–411 10.1038/nrgastro.2017.38 28487545

[CR47] Vermeulen L, De Wilde G, Van Damme P, Vanden Berghe W, Haegeman G (2003) Transcriptional activation of the NF-kappaB p65 subunit by mitogen- and stress-activated protein kinase-1 (MSK1). EMBO J 22:1313–1324 12628924 10.1093/emboj/cdg139 PMC151081

[CR48] Wang Z, Yang X, Liu C, Li X, Zhang B, Wang B, Zhang Y, Song C, Zhang T, Liu M, Liu B, Ren M, Jiang H, Zou J, Liu X, Zhang H, Zhu WG, Yin Y, Zhang Z, Luo J (2019) Acetylation of PHF5A modulates stress responses and colorectal carcinogenesis through alternative splicing-mediated upregulation of KDM3A. Mol Cell 74:1250–1263 31054974 10.1016/j.molcel.2019.04.009

[CR49] Wang YH, Suk FM, Liu CL, Chen TL, Twu YC, Hsu MH, Liao YJ (2020) Antifibrotic effects of a barbituric acid derivative on liver fibrosis by blocking the NF-kappaB signaling pathway in hepatic stellate cells. Front Pharmacol 11:388 32296336 10.3389/fphar.2020.00388 PMC7136425

[CR50] Wang T, Xu R, Qiao F, Du ZY, Zhang ML (2022) Effects of mannan oligosaccharides (MOS) on glucose and lipid metabolism of largemouth bass (*Micropterus salmoides*) fed with high carbohydrate diet. Anim Feed Sci Tech 292:115449

[CR51] Wu X, Gu X, Xue M, Ge C, Liang X (2022) Proteomic analysis of hepatic fibrosis induced by a high starch diet in largemouth bass (*Micropterus salmoides*). Comp Biochem Phys D 43:101007 10.1016/j.cbd.2022.101007 35714397

[CR52] Yeung F, Hoberg JE, Ramsey CS, Keller MD, Jones DR, Frye RA, Mayo MW (2004) Modulation of NF-kappaB-dependent transcription and cell survival by the SIRT1 deacetylase. EMBO J 23:2369–2380 15152190 10.1038/sj.emboj.7600244 PMC423286

[CR53] Zhang SY, Wan D, Zhu MC, Wang GH, Zhang XR, Huang N, Zhang J, Zhang CY, Shang Q, Zhang C, Liu X, Liang FF, Zhang CY, Kong GY, Geng J, Yao LB, Lu SM, Chen YY, Li ZF (2023) CD11b CD43 Ly6C splenocyte-derived macrophages exacerbate liver fibrosis via spleen-liver axis. Hepatology 77:1612–1629 36098707 10.1002/hep.32782 PMC10113005

[CR54] Zhong L, Liu H, Zhang H, Zhang W, Li M, Huang Y, Yao J, Huang X, Geng Y, Chen D, Ouyang P, Yang S, Luo W, Yin L (2022) High starch in diet leads to disruption of hepatic glycogen metabolism and liver fibrosis in Largemouth Bass (*Micropterus salmoides*), Which is mediated by the PI3K/Akt signaling pathway. Front Physiol 13:880513 35677086 10.3389/fphys.2022.880513 PMC9168315

