# Supplementary material for: A novel RELA K119 deacetylation mediated by SIRT7 is a pivotal activator to exacerbate liver inflammation and fibrosis in teleosts
Source: Mar Life Sci Technol. 2025 Apr 21;7(4):856–75. doi: 10.1007/s42995-025-00287-9 (PMC12662976; doi:10.1007/s42995-025-00287-9)
Supplement: Supplementary file 2 — Supplementary file2 (DOC 8490 KB) [file 42995_2025_287_MOESM2_ESM.doc]

**SUPPLEMENTARY INFORMATION**

**A novel RELA K119 deacetylation mediated by SIRT7 is a pivotal activator to exacerbate liver inflammation and fibrosis in teleost**

Xiaoliang Wu ^1^, Xiaofang Liang ^1^, Min Li, Jiacheng Liu, Chunyu Ge, Xiaoze Xie, Jie Wang, Yinhua Zheng, Hao Wang, Xiufeng Wu, Xu Gu, Min Xue ^*^

*National Aquafeed Safety Assessment Center, Institute of Feed Research, Chinese Academy of Agricultural Sciences, Beijing 100081, China*

^1^ These authors contributed equally: Xiaoliang Wu, Xiaofang Liang

* Corresponding author

Co-authors' email addresses and Corresponding author's email addresses

Xiaoliang Wu: 82101201182@caas.cn

Xiaofang Liang: liangxiaofang01@caas.cn

Min Xue *: xuemin@caas.cn

Supplementary Table S1. Formulation and nutrient composition of experimental diets (% of as is)

| **Ingredient (%)** | **ND** | **HCHFD** |
| --- | --- | --- |
| Fish meal | 30 | 30 |
| Cottonseed concentrate protein | 15 | 21 |
| Soybean meal | 14 | 0 |
| Wheat gluten meal | 4 | 4 |
| Clostridium autoethanogenum protein | 3 | 6 |
| Tapioca starch | 11 | 21 |
| Microcrystalline cellulose | 10 | 0 |
| Fish oil | 5 | 5 |
| Soybean oil | 5 | 10 |
| others | 3 | 3 |
| Total | 100 | 100 |
| **Nutrient levels** |  |  |
| Crude protein (%) | 48.1 | 47.5 |
| Crude lipid (%)  Starch (%) | 13.3  9.4 | 18.2  17.9 |
| Ash (%) | 8.9 | 7.9 |
| Gross energy/(MJ/kg) | 20.0 | 21.0 |

Supplementary Table S2. List of primers used

| RT-qPCR | EF1α-F | 5’- TGCTGCTGGTGTTGGTGAGTT-3’ |
| --- | --- | --- |
|  | EF1α-R | 5’- TTCTGGCTGTAAGGGGGCTC-3’ |
|  | TNFα-F | 5’- CTTCGTCTACAGCCAGGCATCG -3’ |
|  | TNFα-R | 5’- TTTGGCACACCGACCTCACC -3’ |
|  | IL-8-F | 5’- CGTTGAACAGACTGGGAGAGATG -3’ |
|  | IL-8-R | 5’- AGTGGGATGGCTTCATTATCTTGT -3’ |
|  | IL-10-F | 5’- CGGCACAGAAATCCCAGAGC -3’ |
|  | IL-10-R | 5’- CAGCAGGCTCACAAAATAAACATCT -3’ |
|  | IL-1β-F | 5’- CGTGACTGACAGCAAAAAGAGG -3’ |
|  | IL-1β-R | 5’- GATGCCCAGAGCCACAGTTC -3’ |
|  | MMP2-F | 5’- ATGGCGCTCCATGTAAATTC -3’ |
|  | MMP2-R | 5’- TTTCAGGGCAGAACCCATAG -3’ |
|  | MMP9-F | 5’- TGCCACCACAGACAACTTTGAC -3’ |
|  | MMP9-R | 5’- CACCACAACTTGCCATCTCCTC -3’ |
|  | BCL-2-F | 5’- TGCCTTTGTGGAGCTGTATG -3’ |
|  | BCL-2-R | 5’- GGAAGAGGAGGAGGAGGATG -3’ |
|  | BAX-F | 5’- AAATGTGGGAGCCAGACATC -3’ |
|  | BAX-R | 5’- AGGCTCCTGGTCTCCTTCTC -3’ |
|  | Acta2-F | 5’- ACGTGTGACTCGGTGTCGTA -3’ |
|  | Acta2-R | 5’- AACCCGGCCTTACACAGAC -3’ |
|  | Col1α1-F | 5’-ATCTGGACCATCTGGCAAAG -3’ |
|  | Col1α1-R | 5’-TCCGGGAATACCAGCACTAC-3’ |
|  | Col1α2-F | 5’- GTCCCCCTGGTAAAGATGGT -3’ |
|  | Col1α2-R | 5’- ACCAGGCAGACCAACAAATC -3’ |
|  | Col2α-F | 5’- GGTACAGAAGCACCGAGAGC -3’ |
|  | Col2α-R | 5’- TGTAGGCAACGCTGTTCTTG -3’ |
|  | P65-F | 5’- GCTGGTGTCTGGTTCATT -3’ |
|  | P65-R | 5’- GCCTCCTCTTCCATCTCT -3’ |
|  | SIRT7-F | 5’- TGAGGACCCCATCTTCAGTC-3’ |
|  | SIRT7-R | 5’- TGCACAGCTACGGCTACATC-3’ |
| EMSA | TNFα-EMSA-F | 5’- TGTACCTCATCTGGTTTTCCTTCAGCTCGG -3’ |
|  | TNFα-EMSA-R | 5’- CCGAGCTGAAGGAAAACCAGATGAGGTACA-3’ |
|  | TNFα-EMSA-F  (mutant competitor) | 5’-TGTACCTCATAGTAGAACTGTTCAGCTCGG -3’ |
|  | TNFα-EMSA-R  (mutant competitor) | 5’-CCGAGCTGAACAGTTCTACTATGAGGTACA -3’ |

Supplementary Table S3. Antibody information

| **Primary Antibodies for Western Blot** | | |
| --- | --- | --- |
| **Antibody name** | **Company** | **Cat No.** |
| Rabbit anti-GAPDH | Zhixian Biotechnology | ABPR001 |
| Rabbit anti-acetyllysine | PTMBio | PTM-105 |
| Rabbit anti-α-SMA | ABclonal | A7248 |
| Rabbit anti-MMP2 | ABclonal | A6247 |
| Rabbit anti-TNFα | HuaAn Biotechnology | N/A |
| Rabbit anti-RELA | HuaAn Biotechnology | N/A |
| Rabbit anti-HA | CST | 3724 |
| Rabbit anti-Histone H3 | ABclonal | A2348 |
| Rabbit anti-GFP | CST | 2956 |
| Rabbit anti-SIRT7 | Solarbio | K008235P |
| **Primary Antibodies for Immunofluorescence** | | |
| **Antibody name** | **Company** | **Cat No.** |
| Rabbit anti-RELA | HuaAn Biotechnology | N/A |
| Rabbit anti-acetyllysine | CST | 9441 |
| Rabbit anti-SIRT7 | Abclonal | A0979 |
| Rabbit anti-α-SMA | ABclonal | A7248 |
| **Primary Antibodies for Co-Immunoprecipitation** | | |
| Rabbit anti-RELA | HuaAn Biotechnology | N/A |
| Rabbit anti-HA | CST | 3724 |
| Rabbit IgG control | Abcam | ab172730 |
|  | | |
| **Secondary Antibodies for Western blot** | | |
| Goat Anti-Rabbit IgG H&L (HRP) | Abcam | ab6721 |
| **Secondary Antibodies for Immunofluorescence** | | |
| Goat anti-Rabbit IgG, Alexa Fluor 555 | Life Technologies | A21428 |
| Goat anti-Rabbit IgG, Alexa Fluor 488 | Life Technologies | A 11008 |
| **Secondary Antibodies for Co-Immunoprecipitation** | | |
| HRP-conjugated AffiniPure Mouse Anti-Rabbit IgG Light Chain | Abclonal | AS061 |
| Goat Anti-Rabbit IgG H&L (HRP) | Abcam | ab6721 |


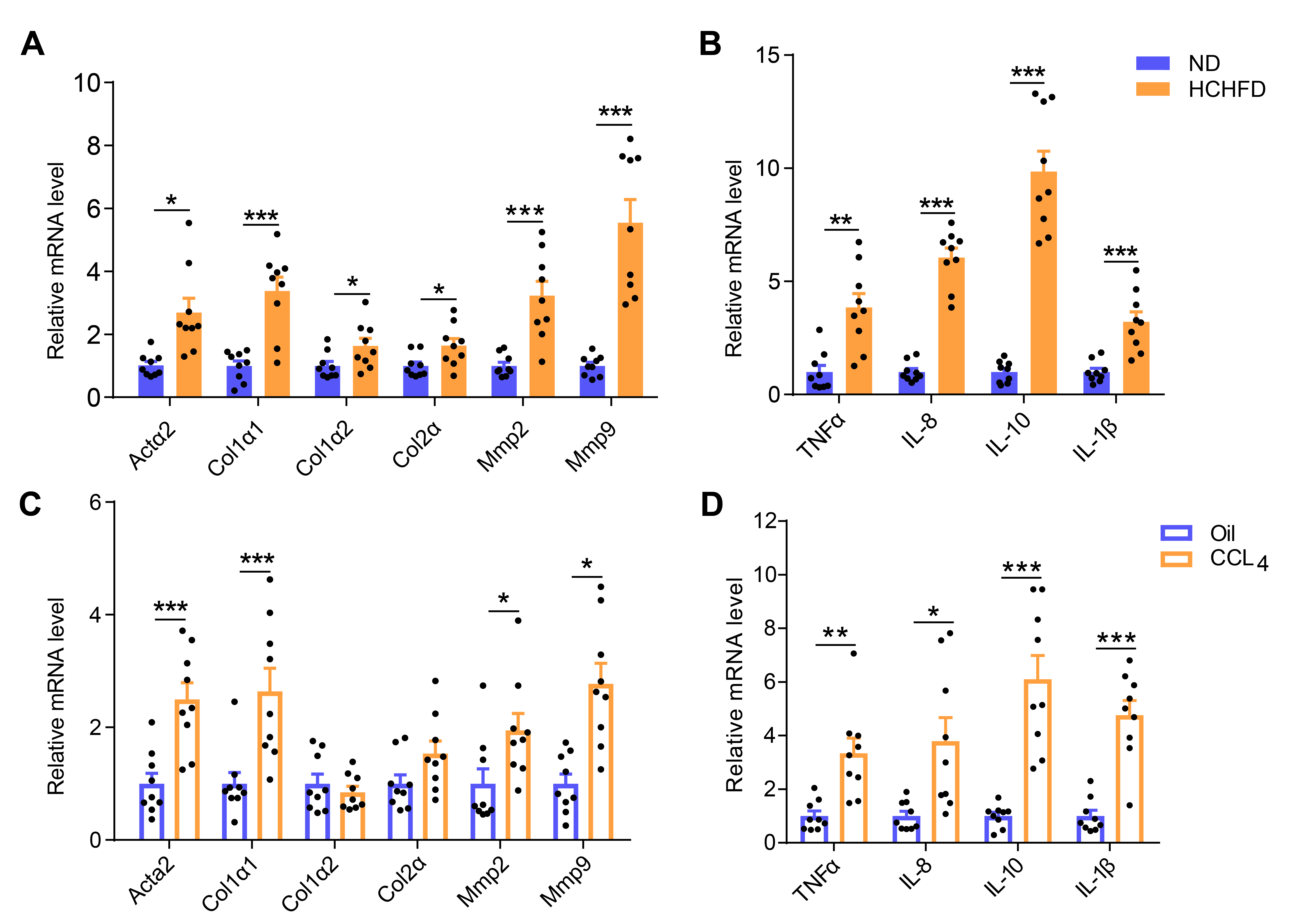


**Supplementary Figure S1 Liver fibrosis was successfully induced by HCHFD and CCL4 administration. A-B** qRT-PCR analysis of the indicated fibrosis related genes (**A**) and inflammation related genes (**B**) in livers induced by ND versus HCHFD (n = 9). **C-D** qRT-PCR analysis of the indicated fibrosis related genes (**C**) and inflammation related genes (**D**) in livers induced by Oil versus CCL_4_ (n = 9). Data were presented as mean±SEM. *P*-values were determined by two-tailed unpaired Student’s t-tests. **p* < 0.05 was considered significant, ***p* < 0.01, ****p* < 0.001.


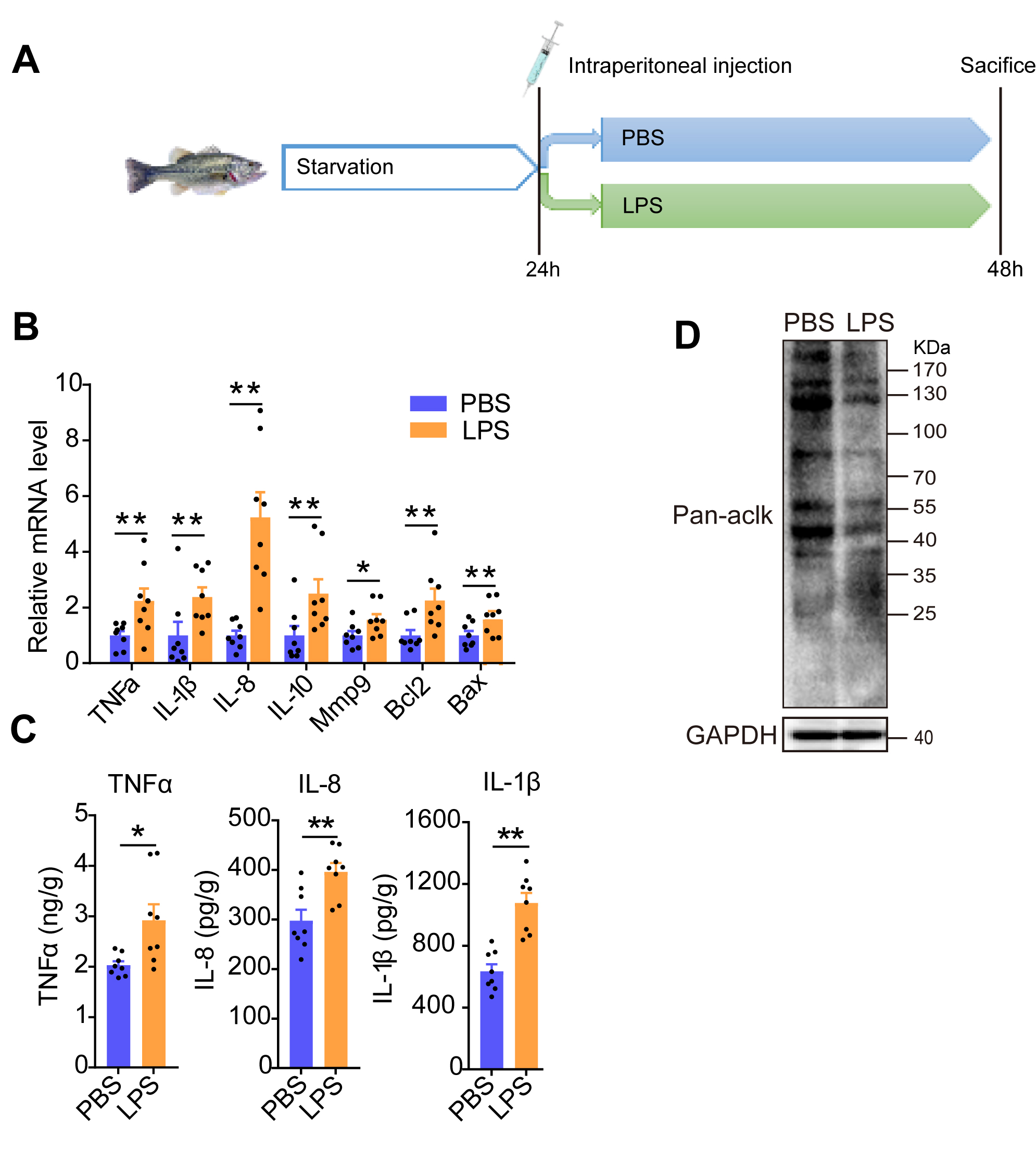


**Supplementary Figure S2 LPS-induced fish model with acute liver inflammation. A** Schematic of experiment design**. B** qRT-PCR analysis of the RELA target genes in livers induced by PBS versus LPS (n = 8). **C** Protein levels of TNFα, IL-1β, and IL-8 in the total protein extracted from livers were assessed by ELISA (n = 8). **D** Representative western blot of the total acetylation in the LPS or PBS-treated livers (n=4). Data were presented as mean±SEM. *P*-values were determined by two-tailed unpaired Student’s t-tests. **p* < 0.05 was considered significant, ***p* < 0.01, ****p* < 0.001.


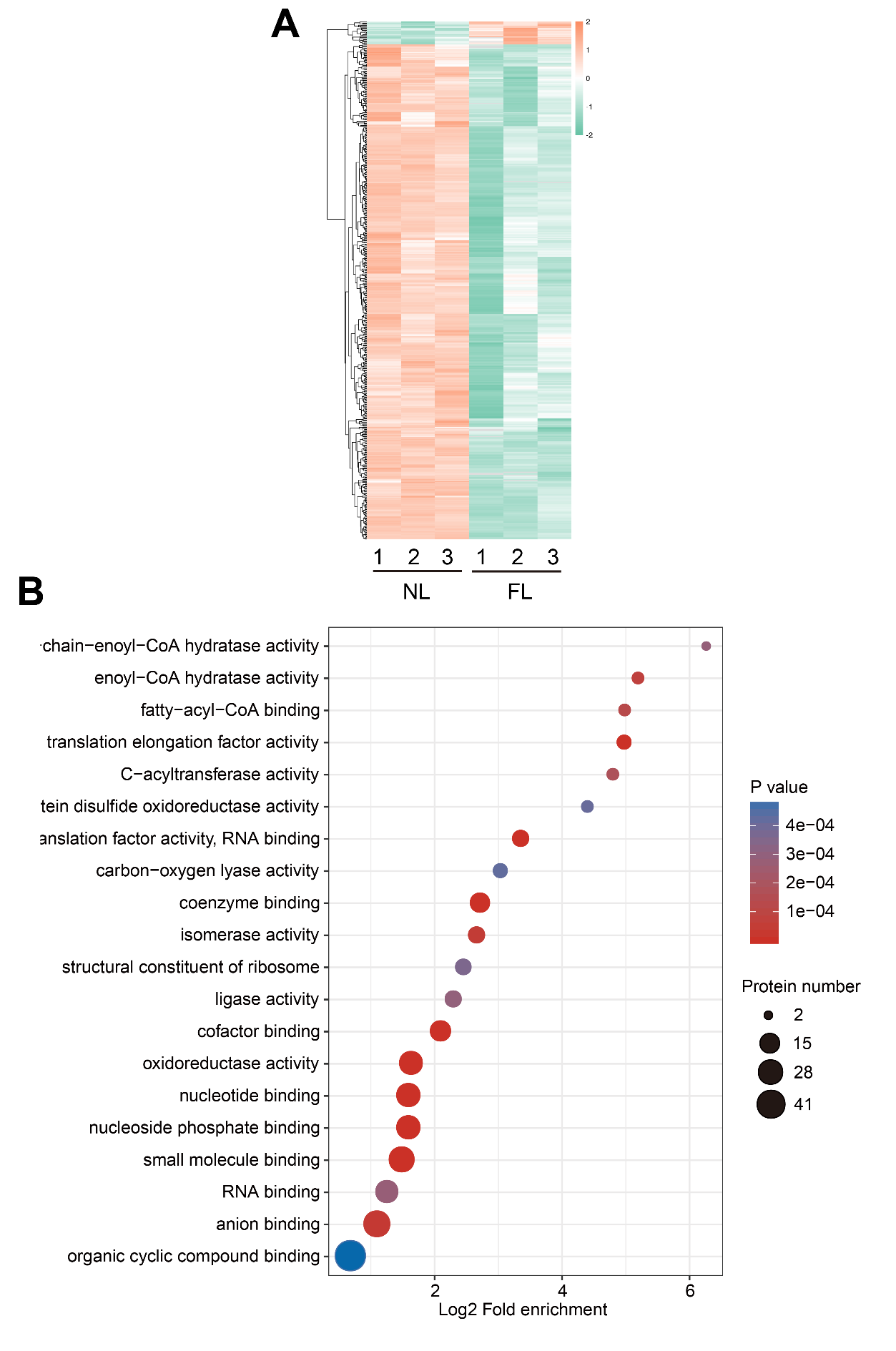


**Supplementary Figure S3 A** Heatmap analysis of differentially acetylated proteins shows clearly distinction between normal livers (NL) and fibrotic livers (FL) induced by ND versus HCHFD. **B** The enrichment analysis of 163 acetylated proteins without changes in total protein levels.


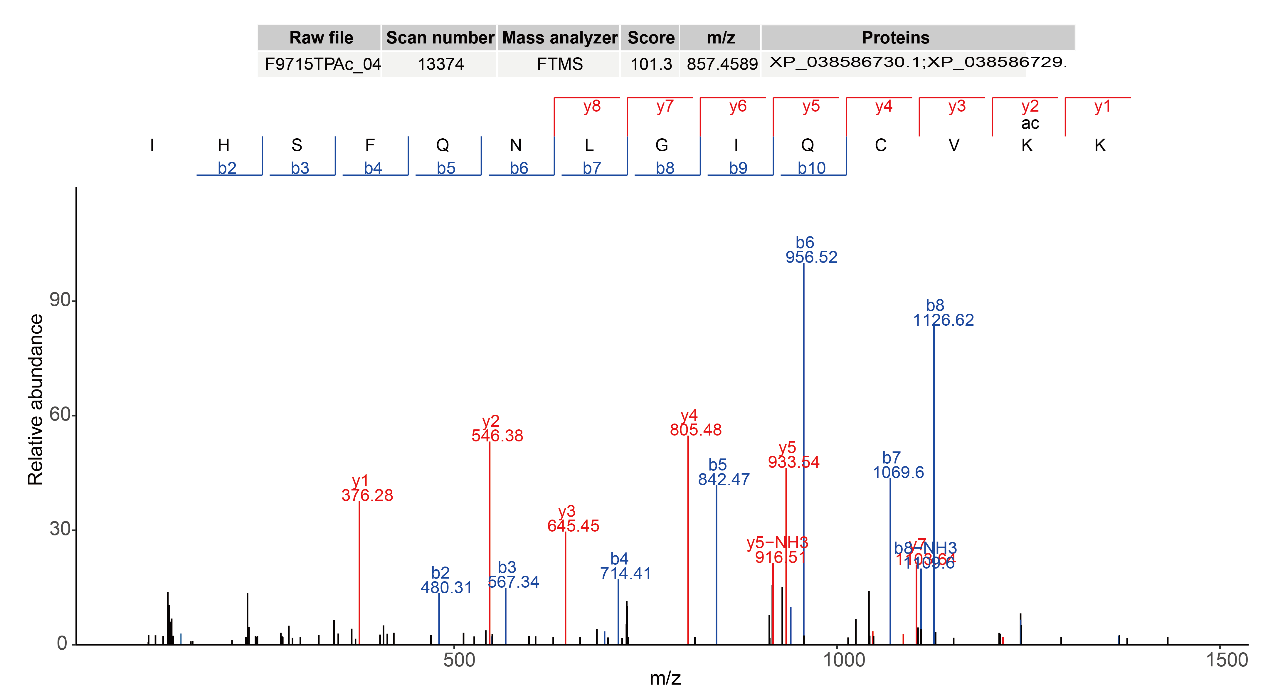


**Supplementary Figure S4** Determination of RELA modification sites by collision-induced dissociation (CID) analysis. The MS/MS spectrum of modified ‘‘IHSFQNLGIQCVK(acetyl) K’’ is shown.


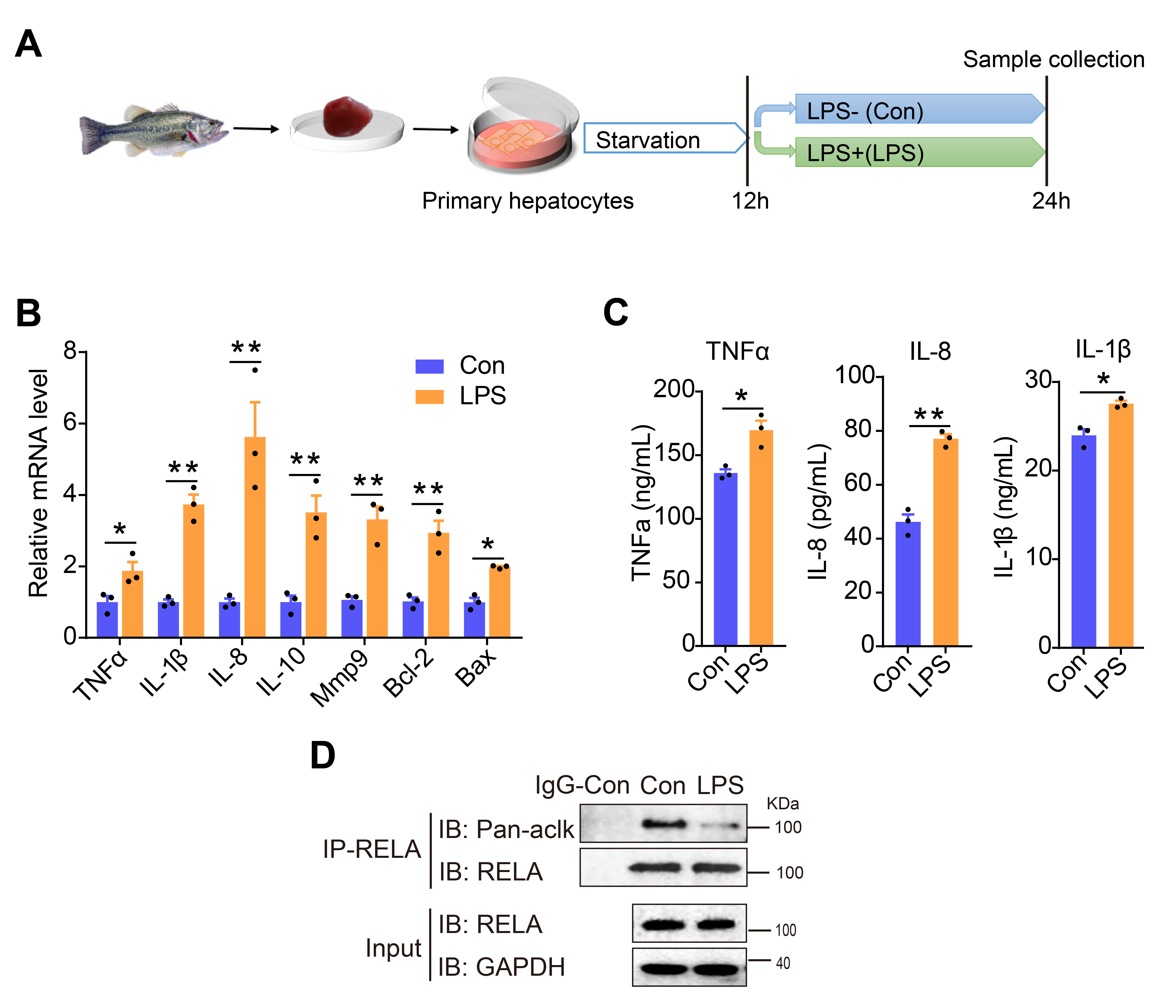


**Supplementary Figure S5 The acetylation of RELA is decreased in primary hepatocytes stimulated with LPS. A** Schematic of experiment design**. B** qRT-PCR analysis of the RELA target genes in primary hepatocytes treated with LPS for 12 hours (n=3). **C** Protein levels of TNFα, IL-1β, and IL-8 in the supernatant treated with LPS for 12 hours were assessed by ELISA (n=3). **D** Representative western blot image for levels of acetylated RELA in primary hepatocytes treated with LPS versus control for 12 hours (n=3). Data were presented as mean±SEM. *P*-values were determined by two-tailed unpaired Student’s t-tests. **p* < 0.05 was considered significant, ***p*< 0.01, ****p* < 0.001.


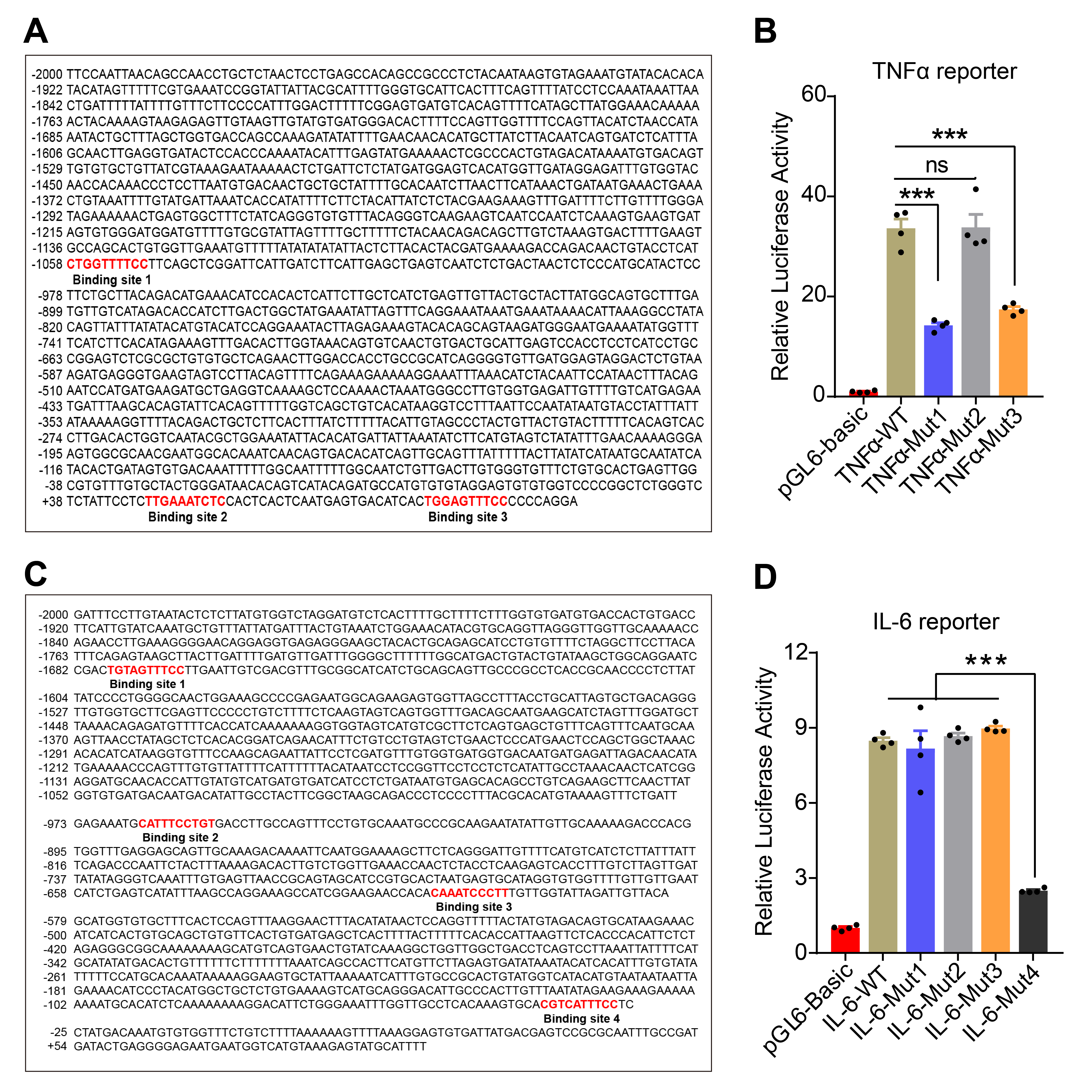


**Supplementary Figure S6 Prediction and determination of RELA binding sites on the TNFα and IL-6 promoter region. A-B** Promoter sequences of TNFα and predicted binding sites of RELA (**A**), and luciferase assays for WT and mutated TNFα promoter in HEK293 cells transfected with WT or mutant TNFα reporter plasmid and RELA expression plasmid for 24 hours(n=4) (**B**). **C-D** Promoter sequences of IL-6 and predicted binding sites of RELA (**C**), and luciferase assays for WT and mutated IL-6 promoter in HEK293 cells transfected with WT or mutant IL-6 reporter plasmid and RELA expression plasmid for 24 hours(n=4) (**D**). Data were presented as mean ± SEM. *P*-values were determined by one-way ANOVA corrected with Tukey’s multiple comparisons tests. **p* < 0.05 was considered significant, ***p* < 0.01, ****p* < 0.001.


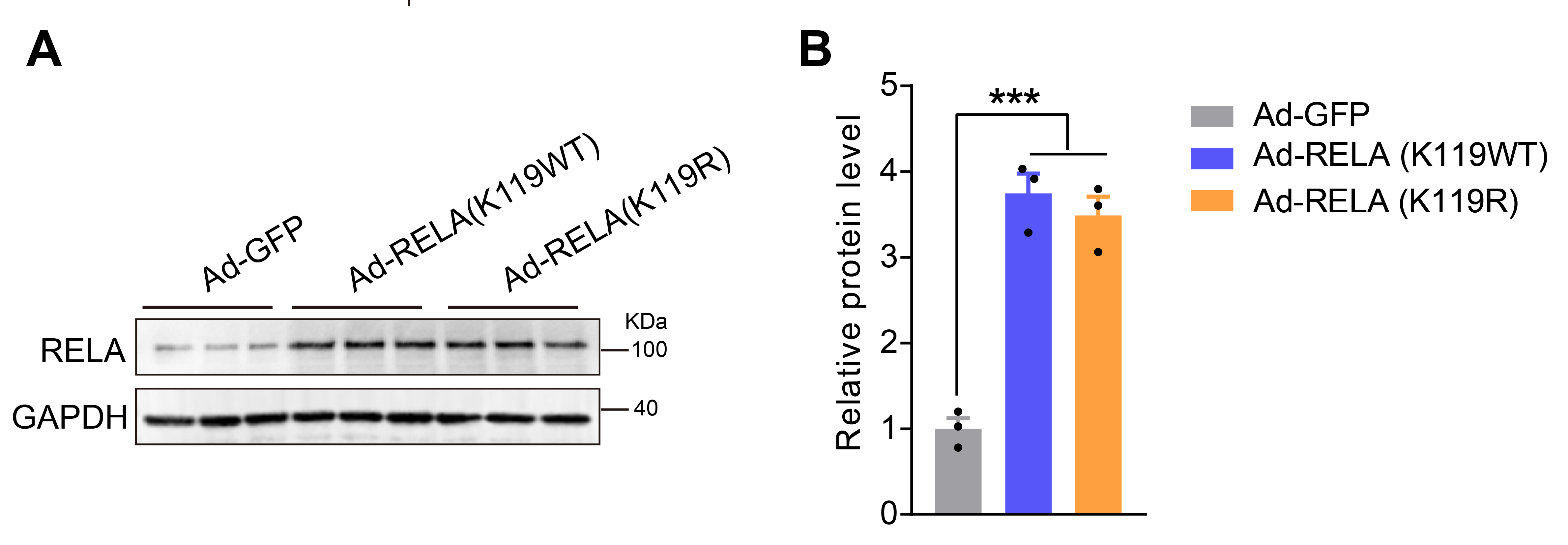


**Supplementary Figure S7 RELA K119WT or K119R were overexpressed in primary hepatocytes by adenoviruses transduction. A-B** Representative western blot of the RELA in primary hepatocytes transduced adenovirus overexpressing RELA K119WT or K119R versus control virus (**A**), and the quantitation of RELA protein levels(**B**) (n=3). Data were presented as mean ± SEM. *P*-values were determined by one-way ANOVA corrected with Tukey’s multiple comparisons tests. **p* < 0.05 was considered significant, ***p* < 0.01, ****p* < 0.001.


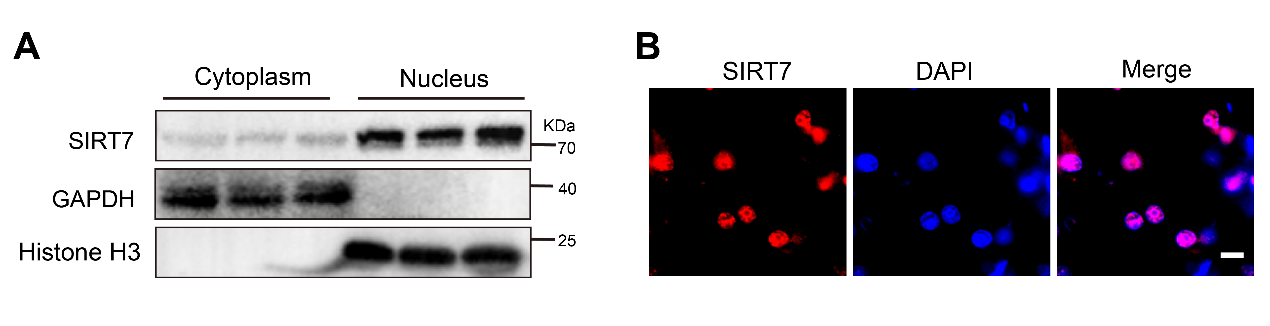


**Supplementary Figure S8 SIRT7 mainly locates in the nucleus in largemouth bass. A** Representative western blot image for Subcellular distribution of exogenous SIRT7 in livers of largemouth bass (n=3). GAPDH and Histone H3 were included as positive control for cytosolic and nuclear protein, respectively. **B** Representative immunofluorescence images of SIRT7 staining for the paraffin sections of liver (n=3). Scale bar for **B**, 8μm.


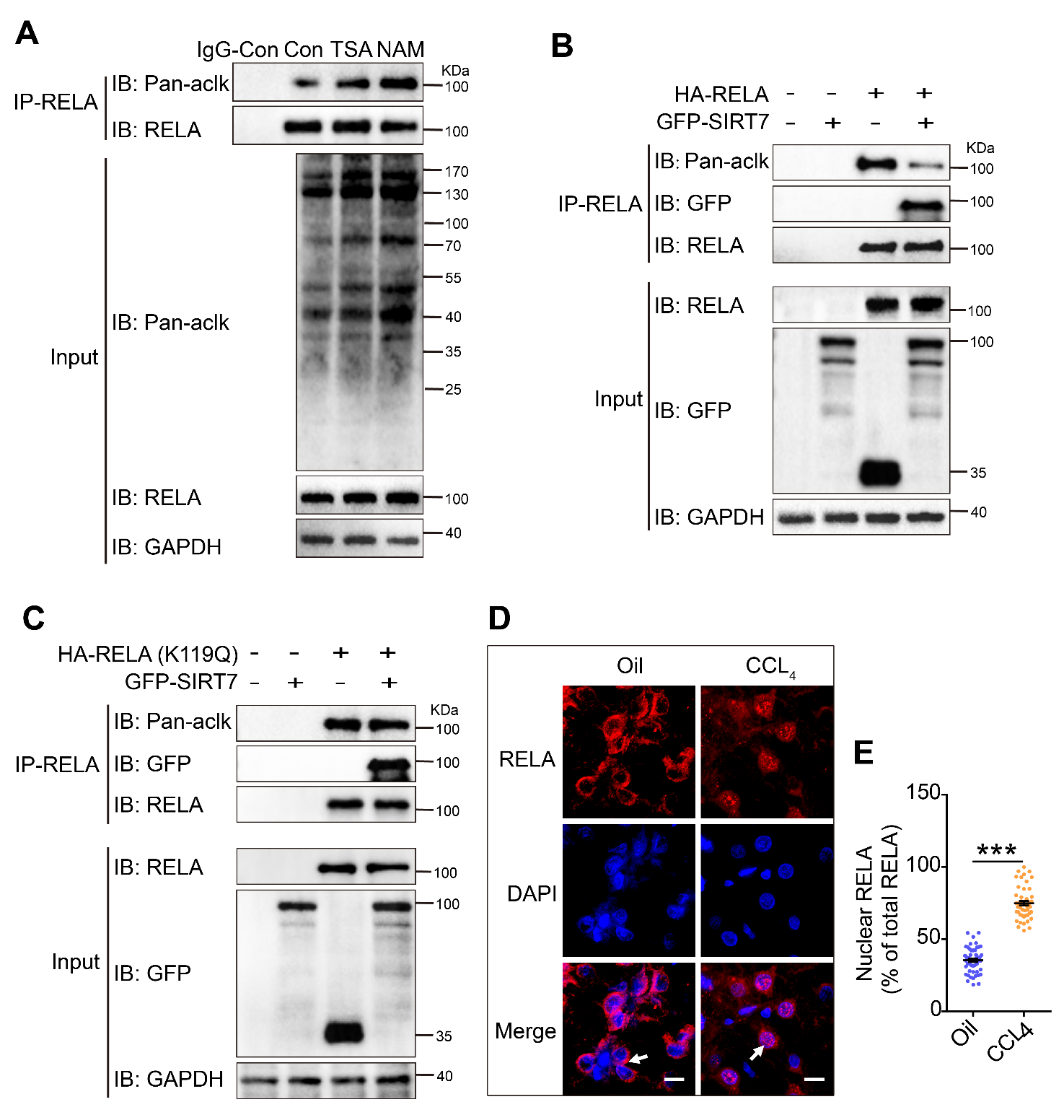


**Supplementary Figure S9 SIRT7 regulates the acetylation of K119 on RELA protein. A** Representative western blot image for the acetylated RELA and total protein from primary hepatocytes treated with HDAC class I and II inhibitor Trichostatin A (TSA) (2μM) and sirtuins inhibitor Nicotinamide (NAM) (10mM) for 24 hours, respectively (n = 3). **B** HEK293T cells were transfected with HA-RELA and GFP-SIRT7 expressing plasmids as indicated for 24 hours prior to protein extraction for RELA co-immunoprecipitation followed by western blotting analysis of the indicated proteins. Representative western blot images for the indicated proteins (n=3). **C** HEK293T cells were transfected with HA-RELA K119Q and GFP-SIRT7 expressing plasmids as indicated for 24 hours prior to protein extraction for RELA K119Q co-immunoprecipitation followed by western blotting analysis of the indicated proteins. Representative western blot images for the indicated proteins (n=3). **D** Representative immunofluorescence images of RELA staining for the paraffin sections from livers induced Oil versus CCL_4_. E Quantification results for panel **D** (n = 50 cells per group). Data were presented as mean ± SEM. *P*-values were determined two-tailed Mann–Whitney U-tests (**E**). **p* < 0.05 was considered significant, ***p* < 0.01, ****p* < 0.001.


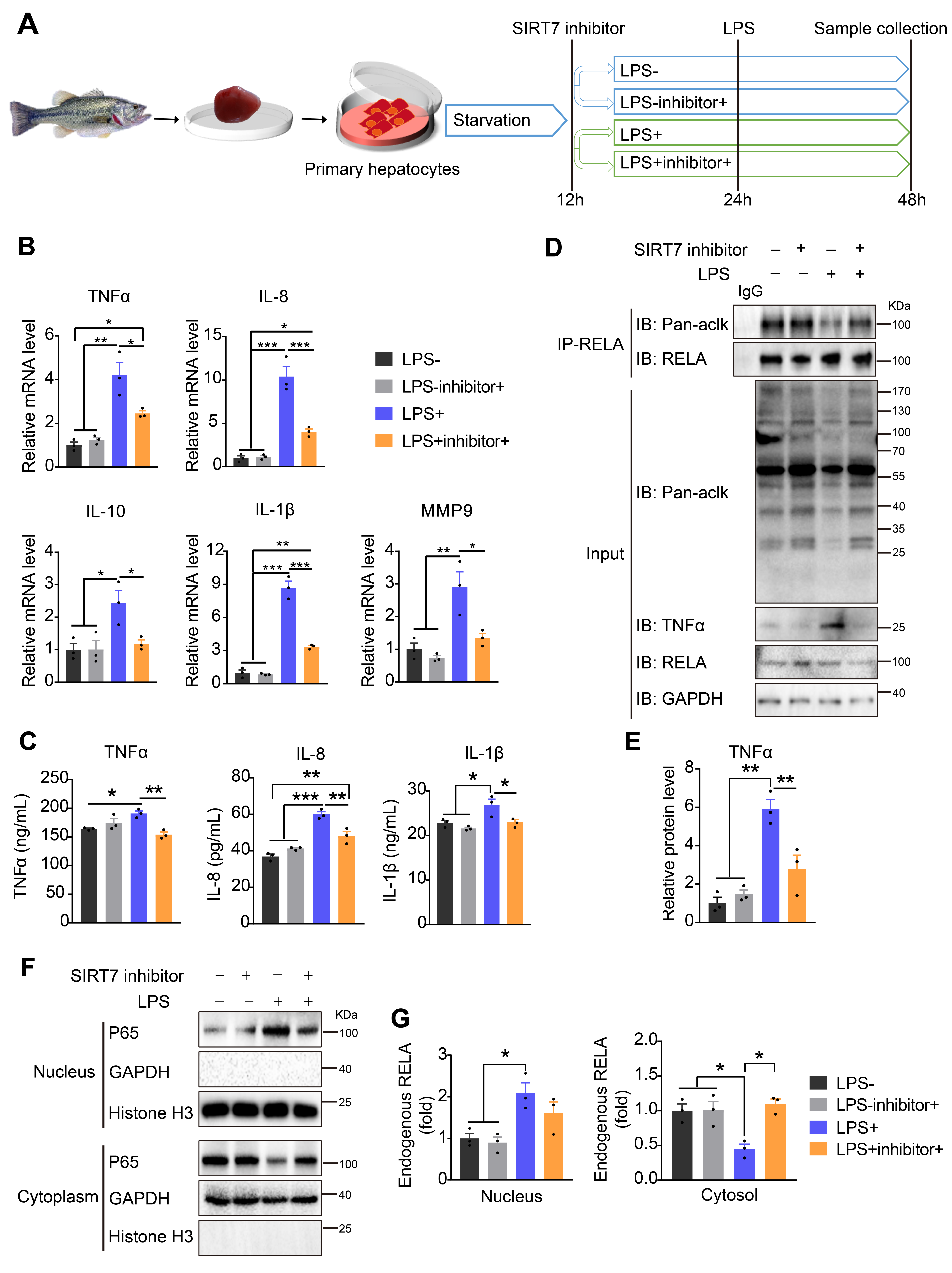
**Supplementary Figure S10 Inhibition of SIRT7 reduces LPS-induced inflammation of primary hepatocytes via reducing the deacetylation of RELA.** Schematic of experiment design. **B** qRT-PCR analysis of the indicated inflammation related genes in primary hepatocytes incubated with SIRT7 inhibitor (10 μM) for 12 hours followed by LPS stimulation for 24 hours (n=3). **C** Protein levels of the indicated proinflammatory factors in the supernatant incubated with SIRT7 inhibitor for 12 hours followed by LPS stimulation for 24 hours (n = 3). **D** Primary hepatocytes were treated with LPS for 12 hours followed by SIRT7 inhibitor (10 μM) for 24 hours prior to protein extraction for RELA co-immunoprecipitation followed by western blotting analysis of the indicated proteins. Representative western blot images for the indicated proteins (n=3). **E** Quantification results of TNFα from panel D**. F** Representative western blot image for subcellular distribution of endogenous RELA in primary hepatocytes under the indicated conditions (n=3). GAPDH and Histone H3 were included as positive controls for cytosolic and nuclear protein, respectively.

**G** Quantitation of nucleus RELA (Left) and cytosolic RELA (Right) immunoblotting signals in panel **F**. Data were presented as mean ± SEM. *P*-values were determined by one-way ANOVA corrected with Tukey’s multiple comparisons test. **p* < 0.05 was considered significant, ***p* < 0.01, ****p* < 0.001.


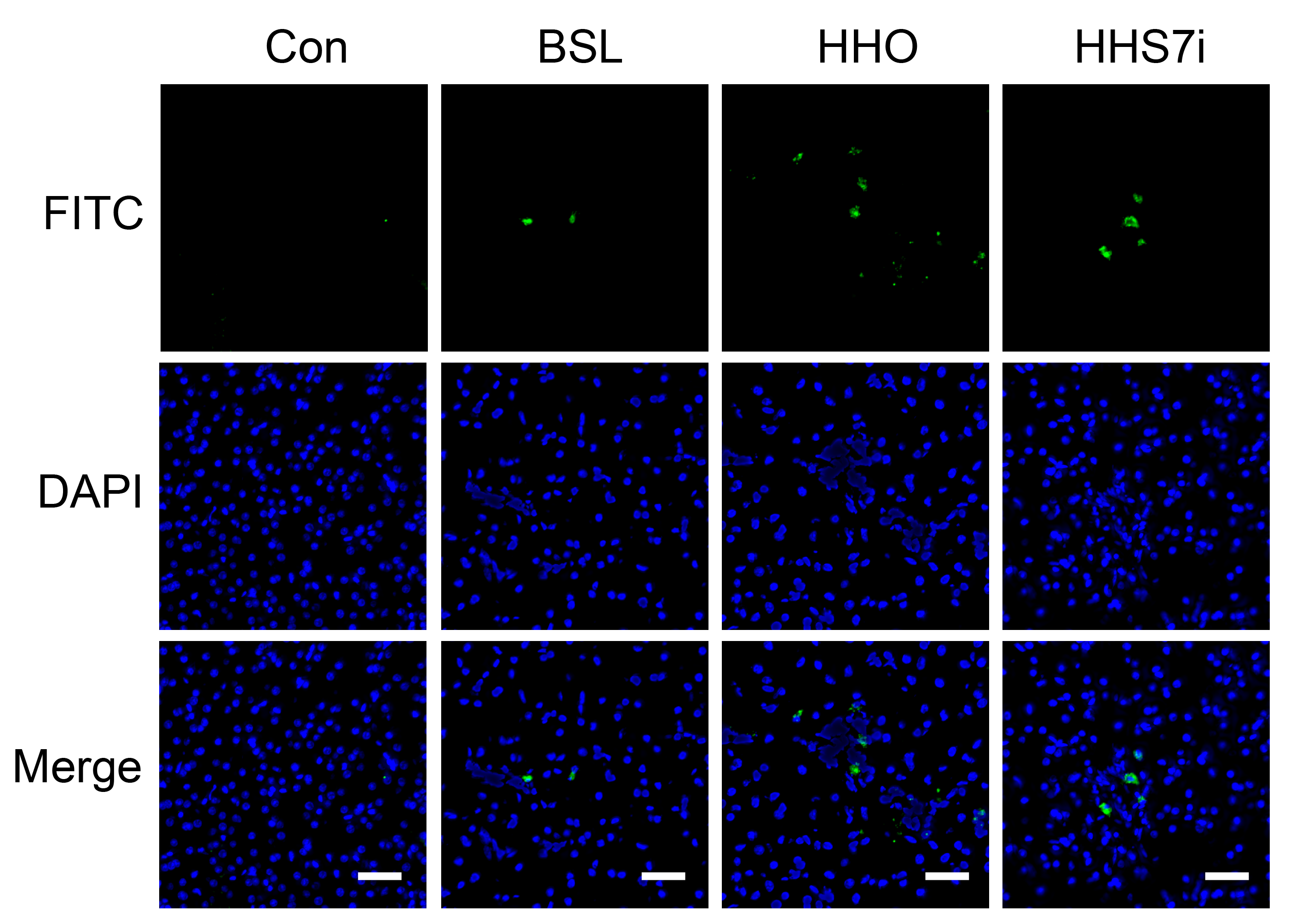


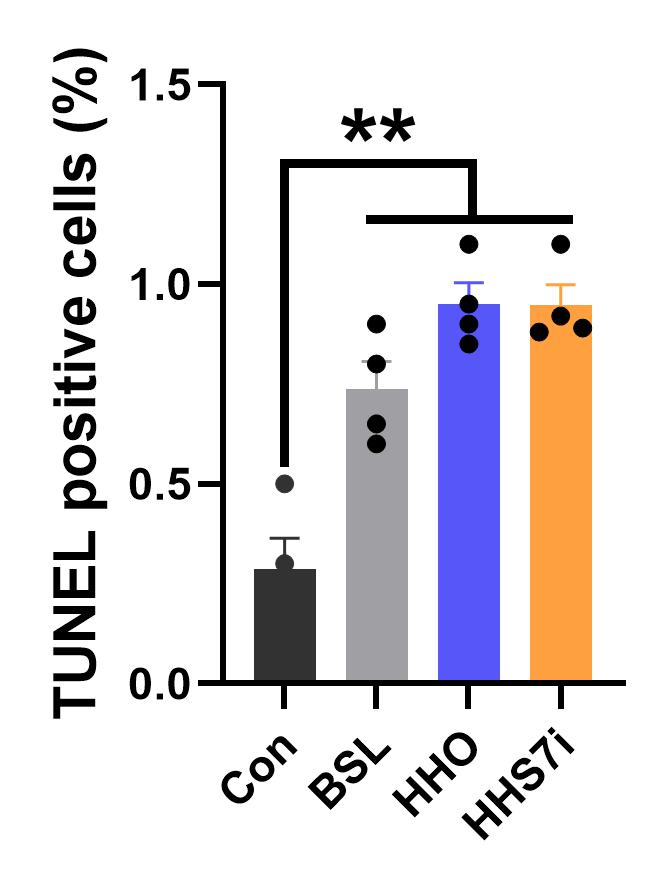


**Supplementary Figure S11 Inhibition of SIRT7 does not trigger mass liver cell apoptosis.** Representative immunofluorescence images of TUNEL staining and statistical analysis for paraffin sections from livers of indicated groups (n=4). Scale bar, 20μm. Data were presented as mean ± SEM. *P*-values were determined by one-way ANOVA corrected with Tukey’s multiple comparisons test. **p* < 0.05 was considered significant, ***p* < 0.01, ****p* < 0.001.
